# Development and validation of a risk model for intracardiac thrombosis in patients with dilated cardiomyopathy: a retrospective study

**DOI:** 10.1038/s41598-024-51745-w

**Published:** 2024-01-16

**Authors:** Yuan Huang, Long-Chang Li, Yu-Xin Li, Chun Gui, Li-Hua Yang

**Affiliations:** 1 Department of Cardiology, Jiangbin Hospital of Guangxi Zhuang Autonomous Region, Nanning, Guangxi China; 2 Department of Cardiology, The First People’s Hospital of Nanning, The Fifth Affiliated Hospital of Guangxi Medical University, Nanning, Guangxi China; 3 Department of Cardiology, The First Affiliated Hospital of Guangxi Medical University, Nanning, 530021 Guangxi China; 4 Guangxi Key Laboratory Base of Precision Medicine in Cardio-Cerebrovascular Diseases Control and Prevention, Nanning, 530021 Guangxi China; 5 Guangxi Clinical Research Center for Cardio-Cerebrovascular Diseases, Nanning, 530021 Guangxi China

**Keywords:** Cardiology, Diseases, Medical research, Risk factors

## Abstract

Intracardiac thrombosis is a severe complication in patients with non-ischemic dilated cardiomyopathy. This study aims to develop and validate an individualized nomogram to evaluate the risk of intracardiac thrombosis in patients with non-ischemic dilated cardiomyopathy. This retrospective study included patients diagnosed with dilated cardiomyopathy at first admission. Clinical baseline characteristics were acquired from electronic medical record systems. Multiple methods were applied to screen the key variables and generate multiple different variable combinations. Multivariable logistic regression was used to build the models, and the optimal model was chosen by comparing the discrimination. Then we checked the performance of the model in different thrombus subgroups. Finally, the model was presented using a nomogram and evaluated from the perspectives of discrimination, calibration, and clinical usefulness. Internal validation was performed by extracting different proportions of data for Bootstrapping. Ultimately, 564 eligible patients were enrolled, 67 of whom developed an intracardiac thrombosis. Risk factors included d-dimer, white blood cell count, high-sensitivity C-reactive protein, pulse pressure, history of stroke, hematocrit, and NT-proBNP in the optimal model. The model had good discrimination and calibration, and the area under the curve (AUC) was 0.833 (0.782–0.884), and the model’s performance in each subgroup was stable. Clinical decision curve analysis showed that the model had clinical application value when the high-risk threshold was between 2% and 78%. The AUC of interval validation (30% and 70% data resampling) was 0.844 (0.765–0.924) and 0.833 (0.775–0.891), respectively. This novel intracardiac thrombosis nomogram could be conveniently applied to facilitate the individual intracardiac thrombosis risk assessment in patients with non-ischemic dilated cardiomyopathy.

## Introduction

Non-ischemic dilated cardiomyopathy (NIDCM) is a heart disease characterized by left ventricular dilatation and systolic dysfunction. Intracardiac thrombosis is one of the complications of NIDCM. Compared with heart failure, NIDCM with intracardiac thrombosis is not uncommon. A study pointed out that the risk of thrombosis in NIDCM patients might be higher than that of patients with ischemic cardiomyopathy (ICM). More than 50% of NIDCM patients had intracardiac thrombi and mural endocardial plaques at autopsy^1^. The instability of blood flow in the heart cavity may increase considering that patients with NIDCM have different degrees of functional mitral regurgitation, thus the thrombosis in the heart cavity would be easy to fall off. If such patients fail to detect and intervene in time, they are often prone to have cardiovascular and cerebrovascular adverse events, which greatly increase the mortality of NIDCM patients. A cohort study of NIDCM and ICM patients combined with left ventricular thrombosis found that despite anticoagulant treatment, patients with left ventricular thrombosis experienced a 20% risk of embolic events and/or death following diagnosis within 1 year^2^. Therefore, we have to pay attention to the prevention of intracardiac thrombosis in patients with NIDCM.

Many risk factors lead to intracardiac thrombosis of patients with NIDCM. Previous studies usually focused on individual factor’s influence and prognostic value. Few studies have comprehensively considered the impact of various factors on intracardiac thrombosis. The Chinese guidelines for the diagnosis and treatment of dilated cardiomyopathy (2018)^3^ recommend that patients who have had mural thrombosis and thromboembolic complications must receive long-term anticoagulation therapy. For patients with atrial fibrillation and the CHA2DS2-VASC score ≥ 2, oral anticoagulation therapy should be considered^4^. However, no corresponding preventive measures have been taken for patients without atrial fibrillation or embolic events. The main reason is that there is currently no effective scoring tool to identify patients at high risk of thrombosis. This study takes a more comprehensive consideration of the impact of various factors on thrombosis, aiming to develop and verify a simple, reliable, and accurate risk-assessment tool for patients with NIDCM to assess the risk of intracardiac thrombosis.

## Methods

### Patients

**Figure 1:**
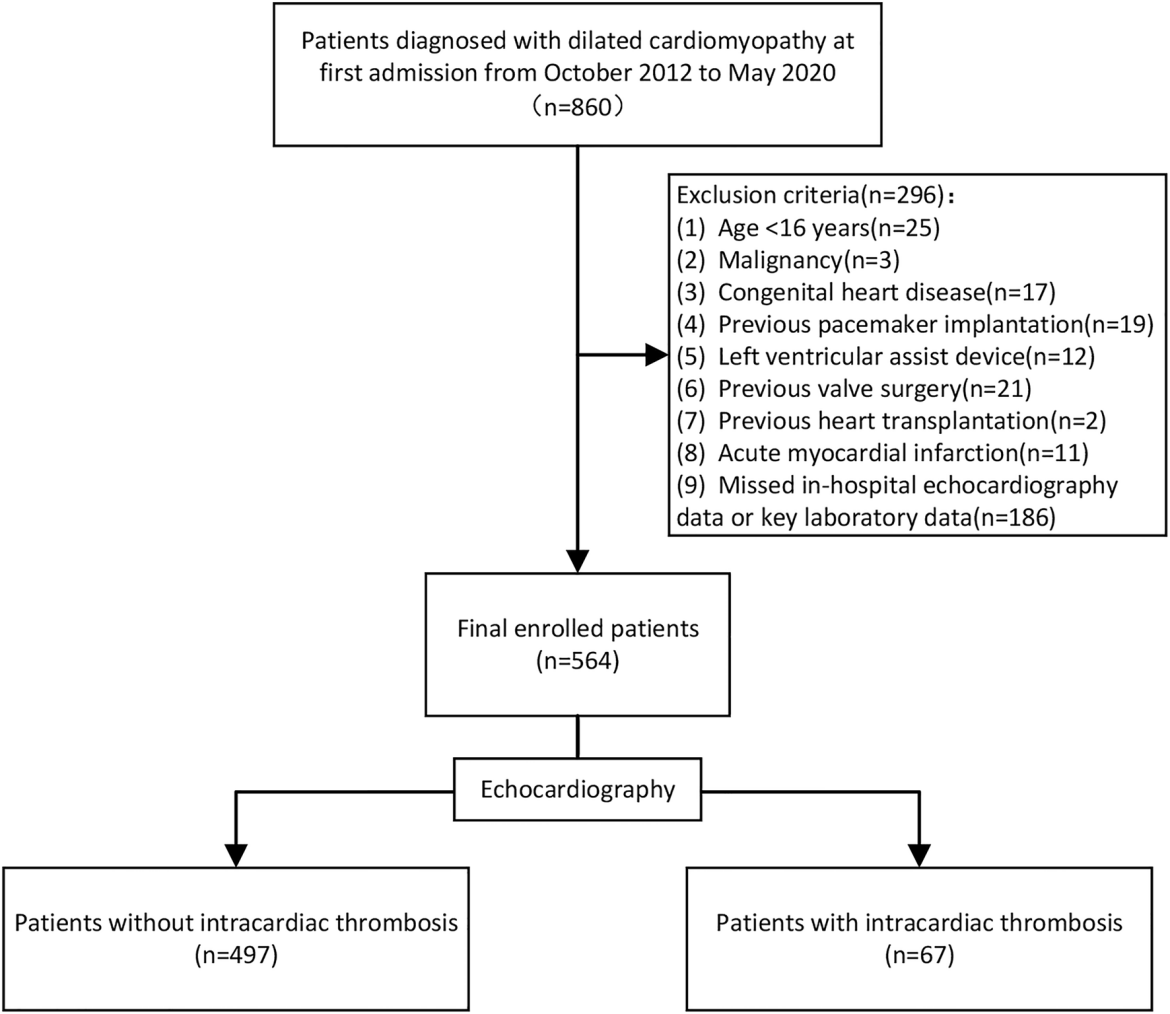
The flow chart of study population enrollment.

This retrospective study consecutively included NIDCM patients at first admission between October 2012 and May 2020 from the First Affiliated Hospital of Guangxi Medical University. As the largest medical referral center with the highest level of diagnosis and therapy in our province, the hospital has approximately 3000 beds and undertakes the task of medical security for tens of millions of people in our province. A total of 860 patients met the diagnostic criteria of NIDCM according to the scientific statement established by the American Heart Association^5^. The exclusion criteria were: (1) age < 16 years, (2) malignancy, (3) congenital heart disease, (4) previous pacemaker implantation, (5) previous left ventricular assist device implantation, (6) previous valve surgery, (7) previous heart transplantation, (8) acute myocardial infarction, (9) missed in-hospital echocardiography data or key laboratory data. Finally, 564 patients were enrolled in the study. In the patients included in this research, the main reason for hospitalization was heart failure, and grade of heart failure III-IV (NYHA) accounted for 79.1%. The diagnosis of thrombus in the heart cavity was based on echocardiography. And the authenticity of the results was further confirmed by at least two senior certified ultrasound physicians. All patients underwent the examination within the first two days of hospitalization. The flow chart of the study population enrollment is shown in Fig. 1.

### Data collection

We collected and analyzed the following variables of the subjects: (1) general information (gender, age of onset, medical history, history of smoking and alcohol, history of stroke, diabetes, atrial fibrillation, pulmonary hypertension); (2) admission physical examination (body mass index, systolic pressure, diastolic pressure, pulse pressure, heart rate); (3) biochemical indicators (the details shown in Table 1); (4) echocardiography indicators (LVEF, LVFS, LVDd, left atrium anteroposterior dimension, left ventricular end-systolic dimension, stroke volume, cardiac output). The information in (1) and (2) were collected within the first 8 h of hospitalization, the data in (3) was collected within the first 24 h of hospitalization, all blood samples were sent to the inspection center of the First Affiliated Hospital of Guangxi Medical University for inspection in time. The data in (4) was collected within the first 48 h of hospitalization. For repeated examinations, only the first results at the time of hospitalization were taken. To make the model more practical, we transformed the measurement data of d-dimer, N terminal pro B type natriuretic peptide (NT-proBNP), white blood cell count (WBC), pulse pressure, and hematocrit into count data based on the optimal cutoff point (Supplementary Table 1) and clinical significance. The high-sensitivity C-reactive protein (hs-CRP) was dichotomized into two groups: “< 1 mg/L” and “≥ 1 mg/L”. Atrial fibrillation (AF) was diagnosed by electrocardiography. Pulmonary hypertension was defined as the pulmonary artery pressure estimated ≥ 30 mmHg based on the tricuspid regurgitation pressure difference.

**Table 1:** Baseline characteristics of the patients with NIDCM between Non-thrombosis and thrombosis groups.

Variables	Total (n = 564)	Non-thrombosis (n = 497)	Thrombosis (n = 67)	Z/χ^2^	P value
General information					
Gender, n (%)				1.03	0.31
Female	124 (22.0)	113 (22.7)	11 (16.4)		
Male	440 (78.0)	384 (77.3)	56 (83.6)		
Age of onset, n (%)				9.65	0.008
< 45 years	170 (30.1)	139 (28)	31 (46.3)		
45–65 years	314 (55.7)	284 (57.1)	30 (44.8)		
≥ 65 years	80 (14.2)	74 (14.9)	6 (9)		
Medical history, n (%)				1.40	0.496
< 1 year	273 (48.7)	238 (48.2)	35 (52.2)		
1–5 years	183 (32.6)	160 (32.4)	23 (34.3)		
≥ 5 years	105 (18.7)	96 (19.4)	9 (13.4)		
Smoking history, n (%)				0	1
No	295 (52.3)	260 (52.3)	35 (52.2)		
Yes	269 (47.7)	237 (47.7)	32 (47.8)		
Drinking history, n (%)				0.24	0.627
No	300 (53.2)	262 (52.7)	38 (56.7)		
Yes	264 (46.8)	235 (47.3)	29 (43.3)		
Hypertension, n (%)				0.26	0.611
No	463 (82.1)	406 (81.7)	57 (85.1)		
Yes	101 (17.9)	91 (18.3)	10 (14.9)		
Pulmonary hypertension, n (%)				0.003	0.958
No	191 (33.9)	169 (34)	22 (32.8)		
Yes	373 (66.1)	328 (66)	45 (67.2)		
Atrial fibrillation, n (%)				0	1
No	454 (80.5)	400 (80.5)	54 (80.6)		
Yes	110 (19.5)	97 (19.5)	13 (19.4)		
History of stroke, n (%)				6.85	0.009
No	509 (90.2)	455 (91.5)	54 (80.6)		
Yes	55 ( 9.8)	42 (8.5)	13 (19.4)		
Diabetes, n (%)				0.01	0.913
No	473 (83.9)	416 (83.7)	57 (85.1)		
Yes	91 (16.1)	81 (16.3)	10 (14.9)		
Grade of heart failure (NYHA), n (%)				0.156	Fisher
I	20 ( 3.5)	17 (3.4)	3 (4.5)		
II	98 (17.4)	92 (18.5)	6 (9)		
III	214 (37.9)	189 (38)	25 (37.3)		
IV	232 (41.1)	199 (40)	33 (49.3)		
Physical examination					
Body mass index (kg/m^2^)	23.0 (20.5, 25.2)	23.0 (20.4, 25.2)	22.9 (20.9, 25.1)	0.04	0.835
Heart rate (times/min)	89.0 (77.0, 102.0)	89.0 (77.0, 101.0)	93.0 (77.0, 105.5)	1.08	0.298
Systolic pressure (mmHg)	112.0 (102.0, 128.0)	114.0 (102.0, 129.0)	110.0 (103.5, 120.5)	2.87	0.091
Diastolic pressure (mmHg)	75.0 (66.0, 86.0)	75.0 (66.0, 86.0)	76.0 (68.0, 87.5)	0.56	0.454
Pulse pressure					
Measurement data (mmHg)	37.0 (29.8, 49.0)	38.0 (30.0, 50.0)	33.0 (27.5, 39.0)	9.70	0.002
Count data (%)				18.75	< 0.001
< 40 mmHg	320 (56.7)	265 (53.3)	55 (82.1)		
≥ 40 mmHg	244 (43.3)	232 (46.7)	12 (17.9)		
Blood biochemical					
NT-proBNP					
Measurement data (pg/mL)	3998(1987, 8325)	3743 (1798, 8285)	6336 (3452, 9132)	11.04	< 0.001
Count data (%)				15.38	< 0.001
< 1800 pg/mL	127 (22.5)	125 (25.2)	2 (3)		
≥ 1800 pg/mL	437 (77.5)	372 (74.8)	65 (97)		
hs-CRP (%)				11.33	< 0.001
< 1 mg/L	118 (20.9)	115 (23.1)	3 (4.5)		
≥ 1 mg/L	446 (79.1)	382 (76.9)	64 (95.5)		
WBC					
Measurement data (× 10^9^/L)	7.5 (6.2, 9.1)	7.3 (6.1, 9.0)	8.1 (7.2, 10.4)	11.73	< 0.001
Count data (%)				11.84	< 0.001
< 10 × 10^9^/L	467 (82.8)	422 (84.9)	45 (67.2)		
≥ 10 × 10^9^/L	97 (17.2)	75 (15.1)	22 (32.8)		
RBC (× 10^12^/L)	4.7 (4.3, 5.1)	4.7 (4.3, 5.1)	4.9 (4.6, 5.3)	3.47	0.062
HB (g/L)	136.0 (125.0, 148.0)	135.0 (125.0, 147.0)	141.0 (131.5, 155.5)	8.94	0.003
Platelet (× 10^9^/L)	197.5 (162.8, 250.0)	201.0 (163.0, 250.0)	186.0 (154.0, 226.0)	2.51	0.113
NEU%	0.6 ± 0.1	0.6 ± 0.1	0.7 ± 0.1	5.26	0.022
NLR	2.6 (1.8, 4.0)	2.6 (1.8, 3.9)	3.3 (1.8, 4.7)	2.61	0.106
RDWCV	0.2 (0.1, 0.2)	0.2 (0.1, 0.2)	0.2 (0.1, 0.2)	6.14	0.013
Hematocrit					
Measurement data	0.4 (0.4, 0.5)	0.4 (0.4, 0.5)	0.4 (0.4, 0.5)	5.11	0.024
Count data (%)				Fisher	< 0.001
< 0.4	192 (34.0)	179 (36)	13 (19.4)		
0.4–0.5	342 (60.6)	300 (60.4)	42 (62.7)		
≥ 0.5	30 ( 5.3)	18 (3.6)	12 (17.9)		
APTT(s)	32.0 (30.2, 33.8)	32.0 (30.3, 33.7)	32.0 (29.6, 34.4)	0.002	0.968
FIB (g/L)	3.8 (3.0, 4.1)	3.8 (3.0, 4.0)	3.5 (2.7, 4.3)	0.25	0.618
TT (s)	12.0 (11.3, 12.5)	12.0 (11.3, 12.5)	12.0 (11.4, 12.8)	0.29	0.59
D-dimer (%)				33.01	< 0.001
Negative (< 450 ng/mL)	315 (55.9)	300 (60.4)	15 (22.4)		
Positive (≥ 450 ng/mL)	249 (44.1)	197 (39.6)	52 (77.6)		
CK (U/L)	91.0 (61.0, 147.0)	88.0 (61.0, 145.0)	110.0 (72.5, 154.5)	2.69	0.101
CK-MB (U/L)	15.0 (11.0, 21.0)	15.0 (11.0, 20.0)	18.0 (11.0, 26.0)	5.56	0.018
LDH (U/L)	261.0 (207.0, 327.5)	252.0 (203.0, 318.0)	299.0 (253.0, 387.5)	21.20	< 0.001
LD1 (U/L)	71.0 (55.0, 95.2)	70.0 (54.0, 91.0)	87.0 (70.5, 113.5)	20.76	< 0.001
α-HBD (U/L)	191.0 (148.0, 230.0)	185.0 (146.0, 226.0)	212.0 (183.5, 276.5)	23.21	< 0.001
TC (mmol/L)	4.2 (3.5, 4.9)	4.2 (3.5, 4.9)	3.9 (3.4, 5.0)	0.17	0.677
TG (mmol/L)	1.0 (0.8, 1.4)	1.1 (0.8, 1.4)	1.0 (0.9, 1.3)	0.65	0.42
HDL (mmol/L)	1.0 (0.8, 1.2)	1.0 (0.8, 1.2)	0.9 (0.7, 1.1)	1.79	0.182
LDL (mmol/L)	2.6 (2.0, 3.2)	2.6 (2.0, 3.2)	2.7 (2.1, 3.1)	0.08	0.784
Hcy (μmol/L)	15.2 (11.9, 18.3)	15.2 (11.9, 18.1)	15.6 (12.6, 19.2)	1.49	0.222
K^+^ (mmol/L)	4.0 (3.7, 4.3)	4.0 (3.7, 4.3)	4.1 (3.7, 4.5)	1.39	0.239
Na^+^ (mmol/L)	139.0 (136.0, 141.0)	139.0 (136.0, 142.0)	138.0 (134.5, 140.0)	7.77	0.005
Cl^−^ (mmol/L)	102.0 (99.0, 106.0)	103.0 (99.0, 106.0)	102.0 (98.0, 104.0)	4.22	0.04
ALB (g/L)	38.2 (35.2, 41.2)	38.3 (35.3, 41.4)	37.4 (33.6, 39.3)	5.08	0.024
GLOB (g/L)	26.2 (23.0, 30.0)	26.2 (23.1, 30.2)	25.0 (22.6, 28.1)	1.80	0.18
A/G	1.5 (1.3, 1.7)	1.5 (1.3, 1.7)	1.4 (1.2, 1.7)	0.35	0.555
AST (U/L)	32.0 (23.0, 47.0)	31.0 (23.0, 46.0)	44.0 (31.0, 55.5)	15.05	< 0.001
ALT (U/L)	31.0 (19.0, 55.0)	30.0 (18.0, 51.0)	40.0 (27.0, 82.5)	15.02	< 0.001
UREA (mmol/L)	7.1 (5.4, 9.1)	7.1 (5.4, 9.0)	7.3 (5.8, 9.6)	0.70	0.401
Creatinine (μmol/L)	93.5 (79.0, 114.0)	93.0 (79.0, 113.0)	99.0 (81.5, 117.5)	1.78	0.183
CysC (mg/L)	1.1 (0.8, 1.3)	1.1 (0.8, 1.3)	1.1 (0.9, 1.4)	0.08	0.772
UA (μmol/L)	495.0 (391.0, 630.0)	485.0 (389.0, 609.0)	604.0 (405.5, 702.0)	8.61	0.003
Echocardiographic					
LAD (mm)	46.0 (41.0, 51.0)	46.0 (42.0, 50.0)	46.0 (41.0, 51.5)	0.001	0.973
LVDd (mm)	69.0 (64.0, 75.0)	69.0 (64.0, 75.0)	70.0 (63.0, 76.0)	0.004	0.951
LVDs (mm)	58.0 (53.0, 63.0)	58.0 (53.0, 63.0)	58.0 (54.0, 64.5)	0.54	0.464
LVFS (%)	16.0 (13.0, 19.0)	16.0 (13.0, 19.0)	15.0 (11.0, 17.5)	5.32	0.021
LVEF (%)	33.0 (26.0, 39.0)	33.0 (27.0, 39.0)	30.0 (23.5, 36.0)	4.74	0.03
SV (mL/B)	80.0 (61.0, 98.0)	80.0 (62.0, 98.0)	80.0 (54.0, 97.5)	0.88	0.349
CO (L/min)	7.1 (5.2, 8.9)	7.1 (5.3, 8.8)	7.2 (4.8, 9.2)	0.002	0.965

*α-HBD* alpha-hydroxybutyrate dehydrogenase, *AST* aspartate aminotransferase, *ALT* alanine aminotransferase, *ALB* albumin, *A/G* ALB/GLOB, *APTT* activated partial thromboplastin time, *CK* creatine kinase, *CK-MB* creatine kinase-MB, *CysC* cystatin c, *CO* cardiac output, *FIB* fibrinogen, *GLOB* globulin, *hs-CRP* high-sensitivity c-reactive protein, *HDL* high density lipoprotein, *HB* hemoglobin, *Hcy* homocysteine, *LDH* lactic dehydrogenase, *LD1* lactic dehydrogenase-1, *LDL* low-density lipoprotein, *LVT* left ventricular thrombosis, *LAD* left atrium anteroposterior dimension, *LVDd* left ventricular end diastolic dimension, *LVDs* left ventricular end systolic dimension, *LVFS* left ventricular fractional shortening, *LVEF* left ventricular ejection fraction, *NT-proBNP* N terminal pro B type natriuretic peptide, *NLR* neutrophil to lymphocyte ratio, *NEU%* percentage of neutrophils, *RDWCV* red blood cell volume distribution width, *RBC* red blood cell count, *SV* stroke volume, *TC* total cholesterol, *TG* triglycerides, *TT* thrombin time, *VIFs* variance inflation factors, *WBC* white blood cell count.

### Statistical analyses

The continuous variables were expressed as median (inter-quartile range) or mean ± standard deviation (SD), and the categorical variables were expressed as percentages. The continuous variables were analyzed using the Mann–Whitney U test or T-test, and the categorical variables were analyzed using the chi-square or Fisher test. We selected variables with a P-value of < 0.1 in the single factor analysis as potential risk factors for further analysis. According to Bayesian Information Criterion (BIC) and adjusted R-square Criterion, we used the best subset approaches to select the variables^6^. After logistic regression, model BIC and model R2 were obtained by using screened variables with minimal BIC and maximal adjusted R-square respectively. Model S/L was constructed based on common variables selected by stepwise selection (Forward and Backward Stepwise) and the least absolute shrinkage and selection operator (LASSO) regression. And the common variables are obtained by combining the variables filtered out by the above two methods and taking the intersection. In the LASSO regression model, variables with nonzero coefficients were selected^7^. The three models’ comparison according to receiver operating characteristic curves (ROC), net reclassification improvement (NRI), and integrated discrimination improvement (IDI) indexes, then treated the optimal model as the baseline model. To verify the stability of the baseline model under different thrombosis distribution conditions, we analyzed the left ventricular thrombosis and left atrial thrombosis as the endpoint events, respectively. Constructed model based on the same variables screened by stepwise selection and LASSO regression. Then compared it with the baseline model. Finally, the baseline model will be presented using a nomogram and evaluated by ROC, calibration curve, clinical decision curve (DCA)^8^, and clinical impact curve^9^. Bootstrapping was used for internal verification (resampling 1000 times using 30% and 70% of the complete data, respectively).

We estimated the sample size for binary outcome events based on 5–10 times the number of variables in the model and the total sample size based on the incidence of events to reach the study size. Statistical analyses were performed using the R software (Version 4.0.4; https://www.r-project.org). A two-tailed analysis with P < 0.05 indicated that the difference was statistically significant.

### Ethics approval and consent to participate

The study protocol was approved by the Human Research Ethics Committee of the First Affiliated Hospital of Guangxi Medical University. Informed consent was waived by the Human Research Ethics Committee of the First Affiliated Hospital of Guangxi Medical University due to retrospective nature of study. All procedures performed in studies involving human participants were in accordance with the ethical standards of the institutional research committee and with the Helsinki declaration and its later amendments or comparable ethical standards.

## Results

### Participants

In this study, a total of 564 NIDCM patients were enrolled, including 440 males and 124 females (mean age 53 ± 14 years). According to the echocardiography, we divided all patients into the non-thrombosis group (497 cases) and thrombosis group (67 cases). Compared with the non-thrombosis group, patients in the thrombosis group had an earlier age of onset and were more likely to have a stroke in the past; the level of pulse pressure was lower. In addition, NT-proBNP, hs-CRP, WBC, NEU%, HB, hematocrit, D-dimer, CK-MB, LDH, LD1, α-HBD, AST, ALT, UA are all higher; Na^+^, Cl^−^, ALB, LVFS, LVEF are lower (P < 0.05) (Table 1). The echocardiographic examination found that the thrombi mainly existed in the apex of the left ventricle, and NIDCM patients with atrial fibrillation were more likely to form in the left atrial appendage. The thrombus distribution is shown in Supplementary Table 2. A large number of patients (n = 186) were excluded from the cohort due to insufficient data, which we collected and compared with the cohort population, see Supplementary Table 3 for details.

### Model development

**Figure 2:**
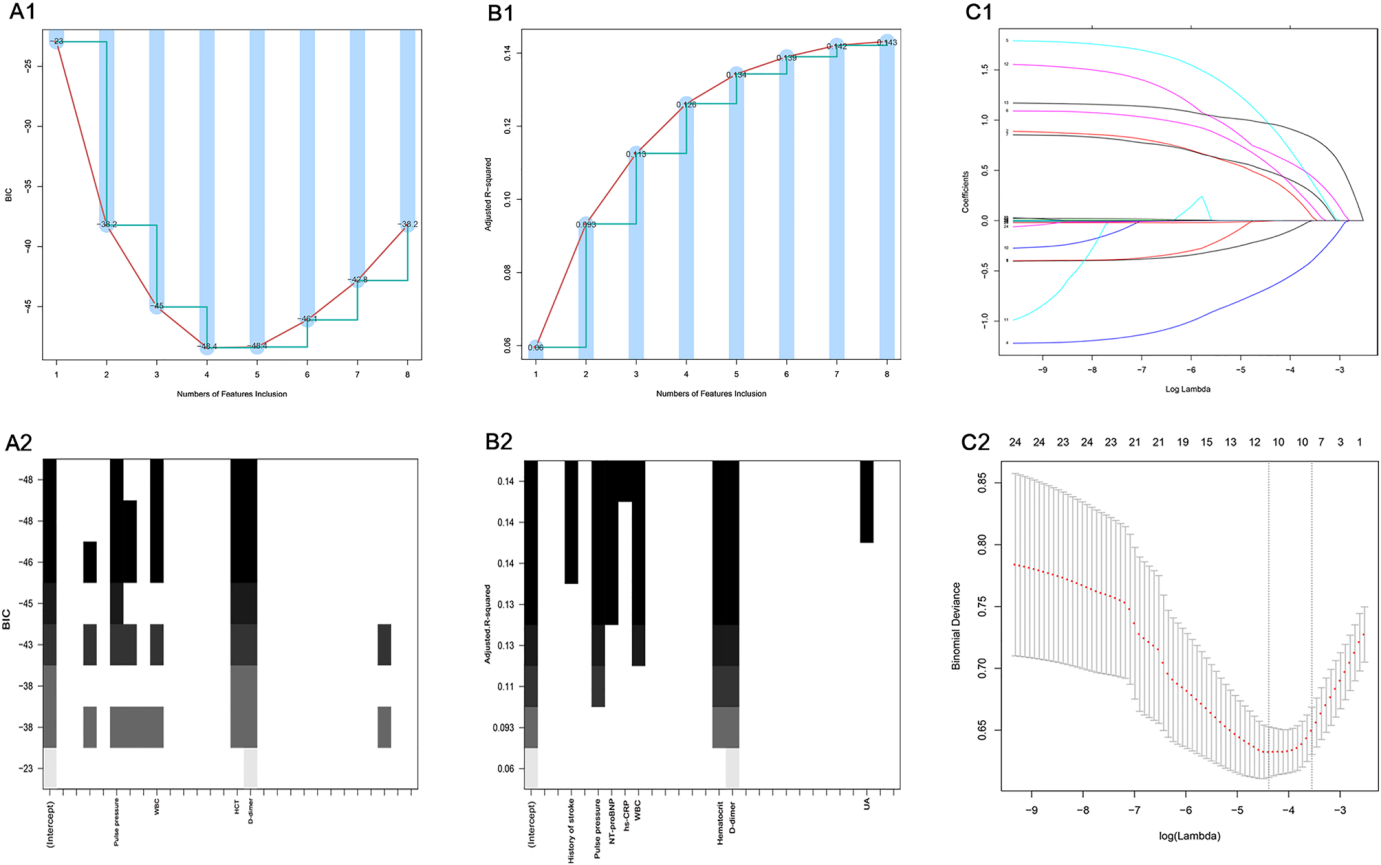
Use several methods for variable screening. (**A1**) The abscissa represents the numbers of variables inclusion, and the ordinate represents the value of BIC; When the number of variables is 4 or 5, the minimum BIC is − 48.4. (**A2**) Specific variables included in the model with minimum BIC. (**B1**) The abscissa represents the numbers of variables inclusion, and the ordinate represents the value of adjusted R-square; when the number of variables is 8, the maximum adjusted R-square is 0.143. (**B2**) Specific variables included in the model with maximum adjusted R-square. (**C1**) LASSO coefficient profiles of the features. A coefficient profile plot was produced against the log (lambda) sequence. (**C2**) Optimal parameter (lambda) selection in the LASSO model used fivefold cross-validation via minimum criteria^40^. The partial likelihood deviance (binomial deviance) curve was plotted versus log (lambda). Dotted vertical lines were drawn at the optimal values by using the minimum criteria and the 1 SE of the minimum criteria (the 1-SE criteria). *BIC* Bayesian Information Criterion, *LASSO* least absolute shrinkage and selection operator, *SE* standard error.

**Figure 3:**
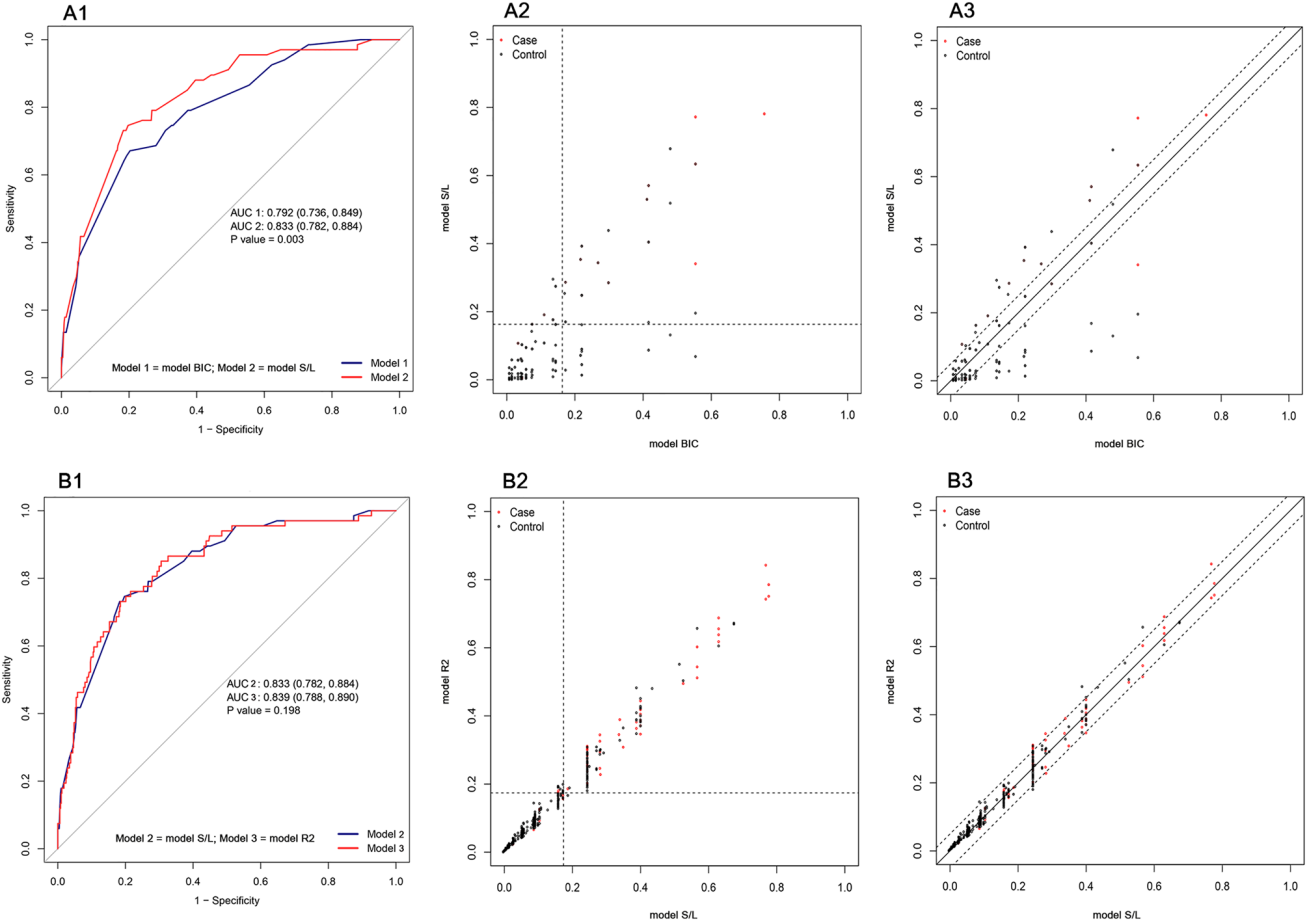
Baseline model selection (comparison between candidate models). (**A1**) Comparison of ROC curves between model BIC and model S/L. (**A2**) Comparison of NRI (Categorical, threshold = 0.163) between model BIC and model S/L. (**A3**) Comparison of NRI (Continuous) between model BIC and model S/L. (**B1**) Comparison of ROC curves between model S/L and model R2. (**B2**) Comparison of NRI (Categorical, threshold = 0.174) between model S/L and model R2. (**B3**) Comparison of NRI (Continuous) between model S/L and model R2. *ROC* receiver operating characteristic curve, *NRI* net reclassification improvement.

We further filter the variables with P < 0.1 in Table 1. First of all, we adopted the best subset approaches, selected the variable combination of minimum BIC: hematocrit, WBC, pulse pressure, d-dimer (Fig. 2A1,A2); and the variable combination of maximum adjusted R squared: hematocrit, WBC, hs-CRP, NT-probNP, pulse pressure, history of stroke, d-dimer, UA (Fig. 2B1,B2). In addition, variables were screened by stepwise regression and LASSO regression, respectively (Fig. 2C1,C2). The same variables screened by the two methods were hematocrit, WBC, hs-CRP, NT-probNP, pulse pressure, history of stroke, and d-dimer. Based on logistic regression, we used the above three groups of variables for modeling named them in sequence as model BIC, model R2, and model S/L, respectively. In the comparison between model BIC and model S/L (Fig. 3A1–A3), AUC_BIC_ = 0.792 (0.736, 0.849), AUC_S/L_ = 0.833 (0.782, 0.884), AUC_S/L_ > AUC_BIC_, P = 0.003; NRI (Categorical) = 0.083, P = 0.02; NRI (Continuous) = 0.492, P < 0.001; IDI = 0.043, P < 0.001. Thus, AUC, IDI, and NRI all indicate that model S/L is superior to model BIC. In the comparison between model S/L and model R2 (Fig. 3B1–B3), AUC_R2_ = 0.839(0.788, 0.890), AUC_R2_ > AUC_S/L_, P = 0.198; NRI (Categorical) =  − 0.002, P = 0.95; NRI (Continuous) = 0.277, P = 0.03; IDI = 0.004, P = 0.36 (Table 2). There is no significant difference in AUC, IDI and NRI between the two models. Hence, the overall comparison shows that model R2 is not superior to model S/L, so we choose the more concise model S/L as the baseline model.

**Table 2:** Comparison of NRI and IDI between models.

Index	Model BIC^a^ VS Model S/L^b^	Model S/L VS Model R2^c^	Baseline model^d^ VS Model S/L-LVT^e^	Baseline model VS Model S/L-LAT^f^
NRI (categorical) [95% CI], p-value	0.083 [0.015–0.150], 0.02	− 0.002 [− 0.062 to 0.058], 0.95	0.021 [− 0.021 to 0.063], 0.33	0.2681 [0.092 to 0.445], < 0.01
NRI +	0.07	0	− 0.019	0.133
NRI −	0.01	0	0.04	0.135
NRI (continuous) [95% CI], p-value	0.492 [0.278–0.705], < 0.001	0.277 [0.026 to 0.527], 0.03	0.489 [0.207 to 0.772], < 0.001	0.744 [0.261 to 1.227], < 0.01
IDI [95% CI], p-value	0.043 [0.026–0.060], < 0.001	0.004 [− 0.004 to 0.011], 0.36	0.021 [0.001 to 0.041], 0.04	0.03 [− 0.042 to 0.102], 0.417

Variables included in each model are as follows: ^a^Model BIC: Hematocrit, WBC, Pulse pressure, D-dimer; ^b^Model S/L: Hematocrit, WBC, hs-CRP, NT-proBNP, Pulse pressure, History of stroke, D-dimer; ^c^Model R2: Hematocrit, WBC, hs-CRP, NT-proBNP, Pulse pressure, History of stroke, D-dimer, UA, ^d^Baseline model: Hematocrit, WBC, hs-CRP, NT-proBNP, Pulse pressure, History of stroke, D-dimer; ^e^Model S/L-LVT: Hematocrit, Age of onset, hs-CRP, NT-proBNP, Pulse pressure, History of stroke, D-dimer; ^f^Model S/L-LAT: AF, AG, WBC, hs-CRP, NT-proBNP, Pulse pressure.

**Figure 4:**
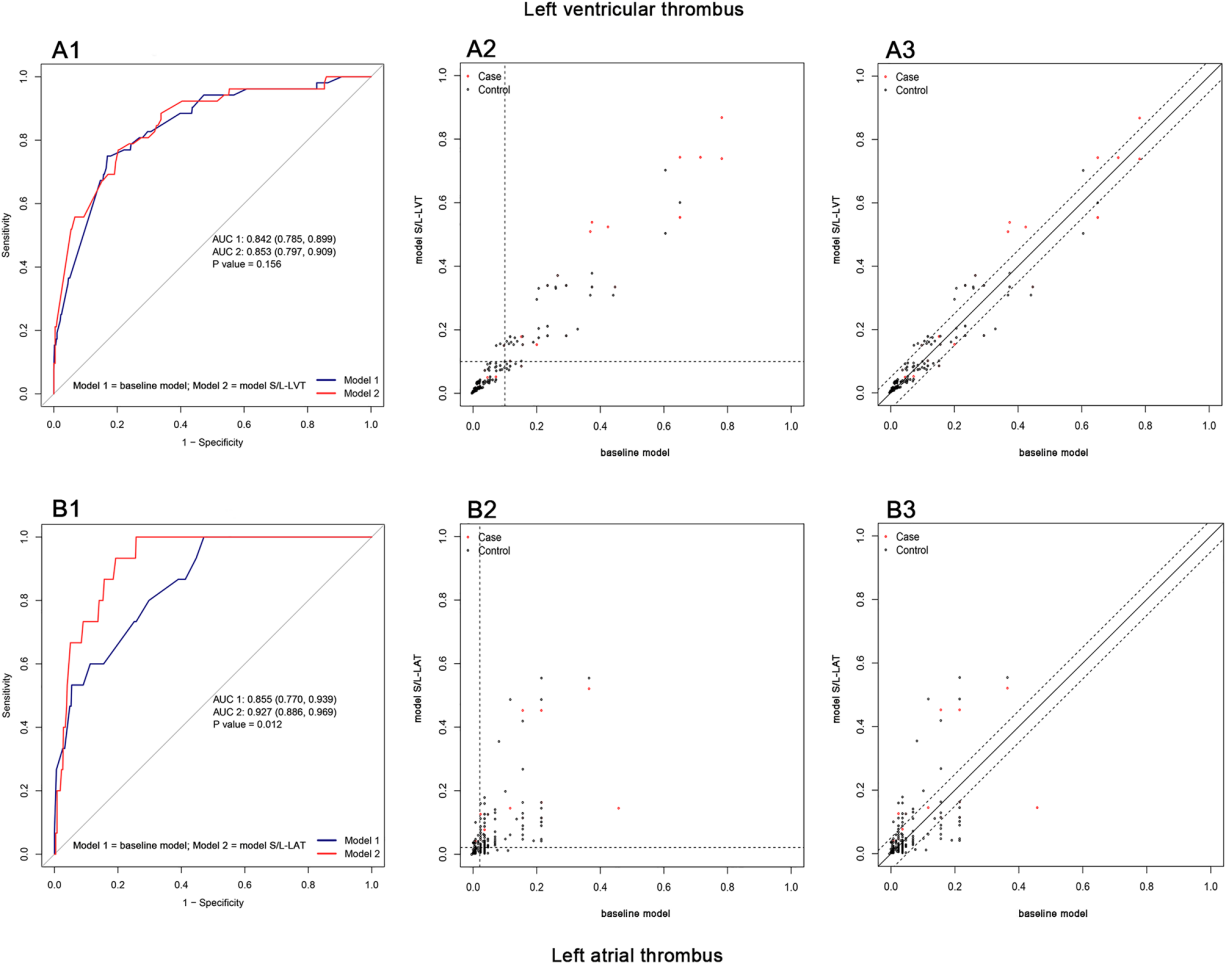
Comparison between the baseline model and the model S/L in different subgroups. In the left ventricular thrombosis subgroup: (**A1**) Comparison of ROC curves between the model S/L-LVT and the baseline model. (**A2**) Comparison of NRI (Categorical, threshold = 0.100) between the model S/L-LVT and the baseline model. (**A3**) Comparison of NRI (Continuous) between the model S/L-LVT and the baseline model. In the left atrial thrombosis subgroup: (**B1**) Comparison of ROC curves between the model S/L-LAT and the baseline model. (**B2**) Comparison of NRI (Categorical, threshold = 0.021) between the model S/L-LAT and the baseline model. (**B3**) Comparison of NRI (Continuous) between the model S/L-LAT and the baseline model. *ROC* receiver operating characteristic curve, *NRI* net reclassification improvement.

Considering that the risk factors of thrombosis in different parts of the heart may be different, the applicability of the baseline model still needs to be further explored. To this end, we performed separate analyses with left ventricular and left atrial thrombosis as endpoints. Variables with a P-value < 0.1 in univariate analysis were included in the stepwise selection and LASSO regression for variable screening; the same variables screened in the two methods were used for modeling. Then the corresponding model S/L-LVT and model S/L-LAT were constructed by logistic regression and compared with the above baseline model, respectively (Fig. 4, Table 2). In the data of left ventricular thrombosis (excluding other cardiac thrombosis data), the screened variables were: age of onset, hematocrit, d-dimer, history of stroke, pulse pressure, NT-proBNP, hs-CRP; The AUC of the model S/L-LVT (AUC = 0.853) was not significantly different from that of the baseline model (AUC = 0.842) (P = 0.156). In the comparison of NRI and IDI, the comprehensive discriminant ability of the model S/L-LVT was only improved by 2.1%, P = 0.04 (Fig. 4A1–A3). In the data of left atrial thrombus (excluding other cardiac thrombus data), the variables of the model S/L-LAT included AF, A/G, WBC, hs-CRP, NT-proBNP, and pulse pressure. The baseline model still showed stable estimation ability (AUC = 0.855), but the model S/L-LAT performed better: AUC = 0.927 (0.886, 0.969), NRI (Categorical) = 0.268, P < 0.01; NRI (Continuous) = 0.744, P < 0.01 (Fig. 4B1–B3). Multivariate logistic regression showed that AF and WBC were the key variables of left atrial thrombosis in the adjusted regression model (Supplementary Table 4). In addition, compared with the baseline model, AF and A/G are non-common variables in the model S/L-LAT, and the other four variables are consistent with the baseline model. Further study on these two special variables showed that when removed “A/G” from the model, AUC = 0.914 (0.865, 0.964), there was no statistical difference between the two models (P = 0.308) (Supplementary Fig. 1). When removed “AF” from the model, AUC = 0.859 (0.768, 0.950), and the comparison between the two models showed a statistical difference (P = 0.031), suggesting that AF is an important risk factor for left atrial thrombosis (Supplementary Fig. 2). Since AF is a well-known cause of thrombosis, it may confound the findings. Therefore, we excluded patients with AF and repeated the above analysis. The same variables screened by stepwise regression and LASSO regression were hematocrit, hs-CRP, NT-probNP, pulse pressure, history of stroke, and d-dimer. The model S/L-NAF has been constructed accordingly and compared with the baseline model. The results showed that there was no statistical difference between the model S/L-NAF and the baseline model in AUC, IDI, and NRI (Supplementary Table 5). The baseline model still demonstrated excellent discrimination and calibration in this population (Supplementary Figs. 3, 4).

**Figure 5:**
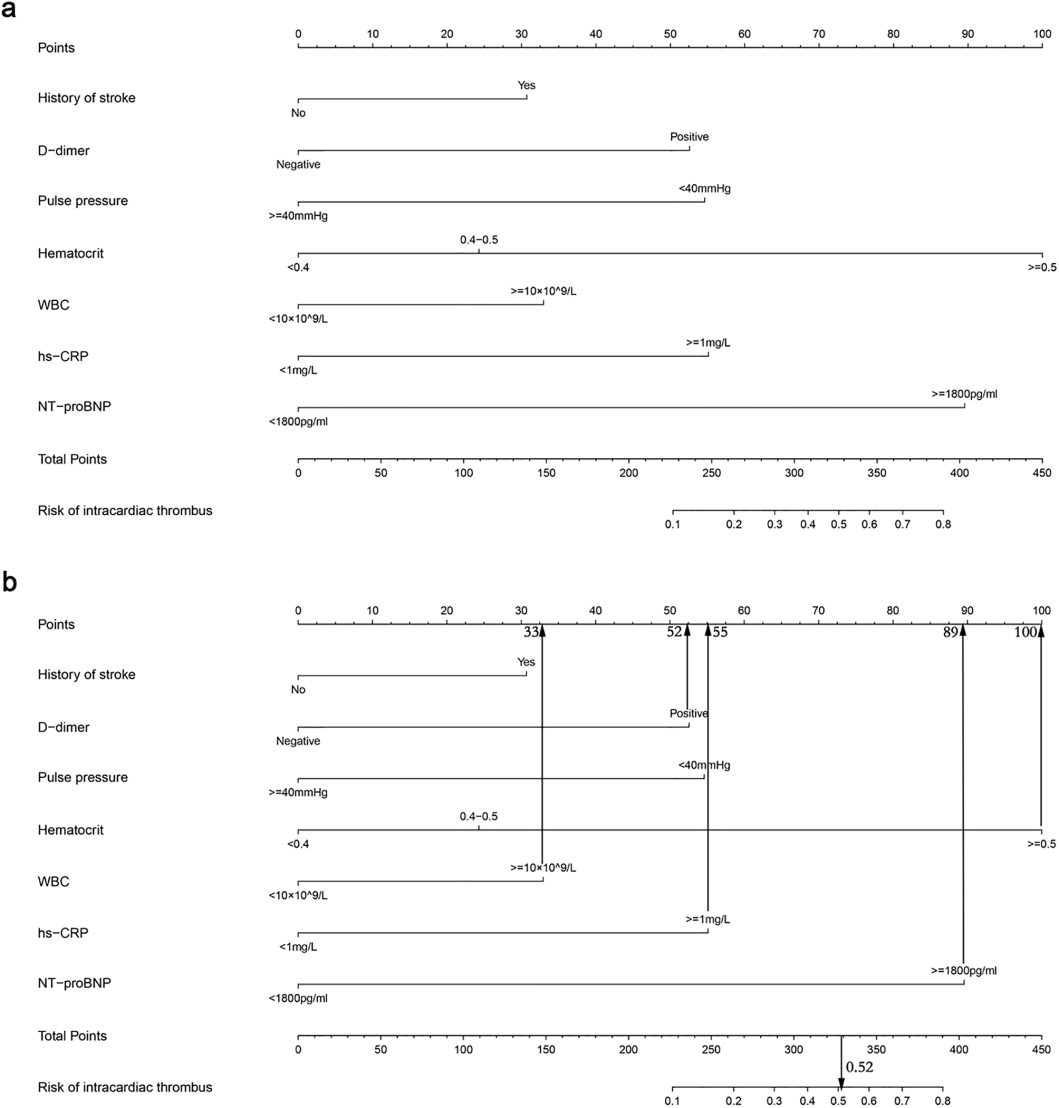
The final model is presented via nomogram. (**a**) Developed intracardiac thrombosis nomogram. (**b**) Example nomogram for risk of intracardiac thrombosis in a NIDCM patient.

We treated the baseline model as the final model based on the above results. Collinearity diagnosis was made for seven variables in the model, and the variance inflation factors were 1.057, 1.154, 1.103, 1.100, 1.053, 1.077, and 1.037, indicating that there was no multicollinearity among the seven variables. The parameters of each variable in the model are shown in Table 3. Multivariate logistic regression analysis revealed that d-dimer [odds ratio (OR) 3.16, 95% confidence interval (CI) 1.65–6.07, P = 0.001], WBC (OR 2.06, 95% CI 1.08–3.92, P = 0.028), NT-proBNP (OR 7.11, 95% CI 1.62–31.24, P = 0.009), pulse pressure (OR 0.3, 95% CI 0.15–0.63, P = 0.001), and hematocrit (≥ 0.5) (OR 8.94, 95% CI 3.08–25.94, P < 0.001) were independently associated with intracardiac thrombus in patients with NIDCM. The nomogram was constructed based on the seven risk factors (Fig. 5a). The interpretation of the nomogram is as follows: For example, one patient with NIDCM had no history of stroke (0 points), with a pulse pressure of 45 mmHg (0 points), and biochemical examination showed that D-dimer was positive (52 points), the WBC was 12 × 10^9^/L (33 points), hs-CRP was 8 mg/L (55 points), hematocrit was 0.55 (100 points), and NT-proBNP was 3600 pg/mL (89 points). The cumulative score of the above risk indicators was 0 + 0 + 52 + 33 + 55 + 100 + 89 = 329, and the corresponding estimated risk of intracardiac thrombosis was 0.52 (52%) (Fig. 5b).

**Table 3:** Logistic regression results of the final model.

Variable	Unadjusted OR (95% CI)	Unadjusted P-value	Adjusted OR (95% CI)	Adjusted P-value
(Intercept)	0.12 (0.09–0.16)	< 0.001	0 (0–0.02)	< 0.001
D-dimer	5.28 (2.89–9.64)	< 0.001	3.16 (1.65–6.07)	0.001
WBC	2.75 (1.56–4.84)	< 0.001	2.06 (1.08–3.92)	0.028
NT-proBNP	10.92 (2.64–45.23)	0.001	7.11 (1.62–31.24)	0.009
Pulse pressure	0.25 (0.13–0.48)	< 0.001	0.3 (0.15–0.63)	0.001
hs-CRP	6.42 (1.98–20.83)	0.002	3.35 (0.97–11.51)	0.055
History of stroke	2.61 (1.32–5.16)	0.006	1.96 (0.88–4.38)	0.101
Hematocrit (0.4–0.5)	1.93 (1.01–3.69)	0.047	1.7 (0.85–3.43)	0.136
Hematocrit (≥ 0.5)	9.18 (3.65–23.09)	< 0.001	8.94 (3.08–25.94)	< 0.001

Final model: Probability (intracardiac thrombus) = 1/1 + exp − [− 5.965 + (D-dimer × 1.152) + (WBC × 0.722) + (NT-proBNP × 1.962) − (Pulse pressure × 1.196) + (hs-CRP × 1.208) + (History of stroke × 0.673) + (Hematocrit^(0.4–0.5)^ × 0.532)/(Hematocrit^(≥0.5)^ × 2.191)].

### Model validation

**Figure 6:**
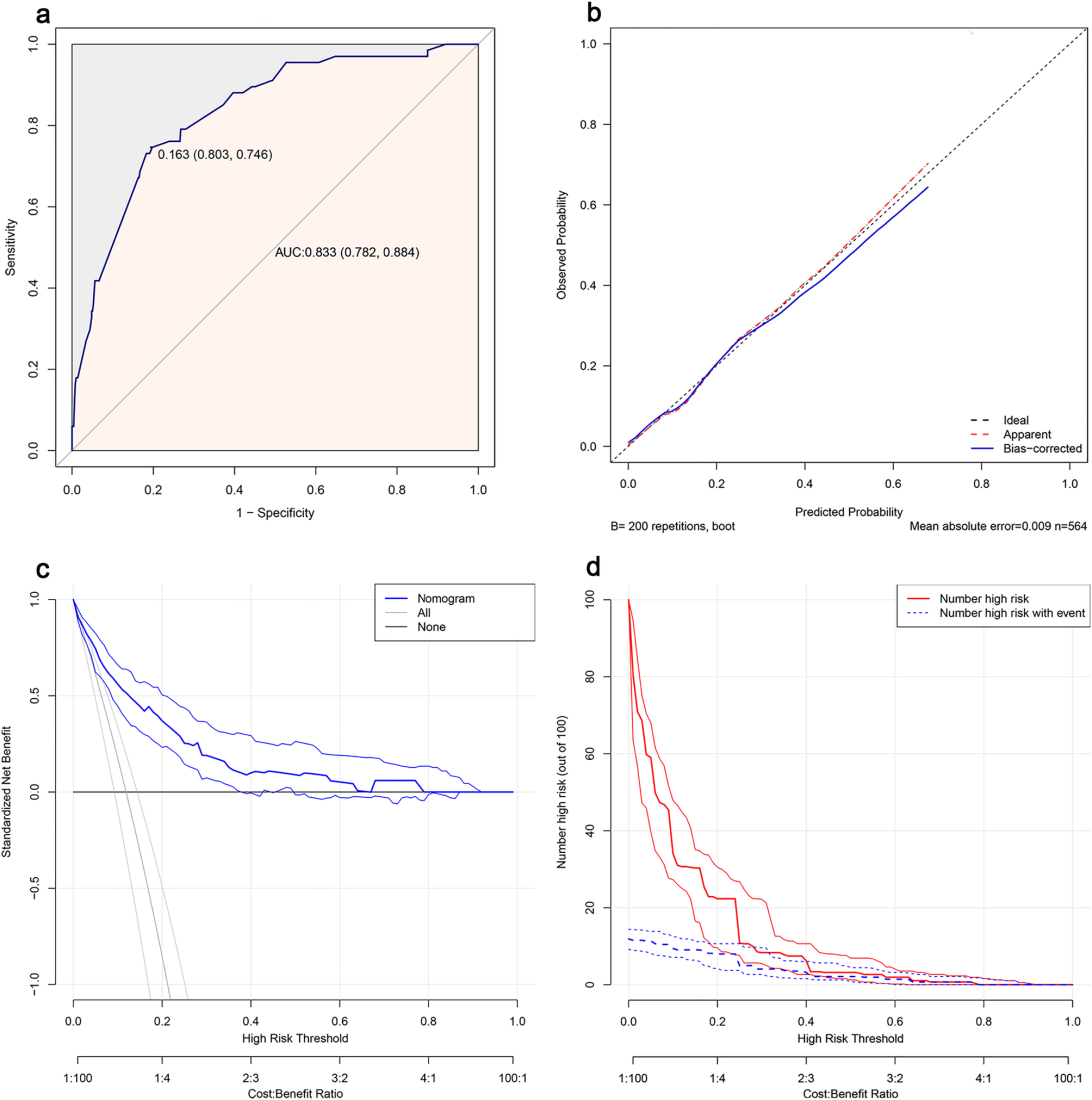
The performance of the final model. (**a**) ROC curve for the final model. (**b**) Calibration curve for the final model. The x-axis represents the estimated intracardiac thrombosis risk. The y-axis represents the actually observed probability of intracardiac thrombosis. The diagonal dotted line represents a perfect estimation by an ideal model. The pink dotted line and the blue solid line represent the performance of the model before and after calibration, respectively. The closer to the diagonal, the better the calibration of the model. (**c**) Decision curve analysis for the final model. The y-axis measures the standardized net benefit. The blue line represents the intracardiac thrombosis risk model and its 95% CI. The thin solid line represents the assumption that all patients occur intracardiac thrombosis (All patients undergo intervention). The thick solid line represents the assumption that no patients occur intracardiac thrombosis (no patients undergo intervention). The decision curve shows that if the threshold probability is in the range of 2% to 78%, using this model to estimate intracardiac thrombosis risk adds more benefit than the intervention-none scheme or intervention-all-patients scheme. (**d**) Clinical impact curve for the final model. The solid red line represents the estimated number of people and 95% CI judged as high risk by the model at different risk thresholds. The dotted blue line represents the actual number of high-risk people and 95% CI at different risk thresholds. *CI* confidence interval.

The validation of the model was based on discrimination and calibration. As shown in Fig. 6, the AUC of the model was 0.833 (95% CI 0.782–0.884), and at the optimal cutoff point, the sensitivity and specificity of the model were 0.803 and 0.746, respectively (Fig. 6a). In addition, we performed the Hosmer–Lemeshow test with a P-value of 0.351 (P > 0.05). And the calibration curve showed that the solid line of the model was close to the dotted line on the diagonal, suggesting good consistency (Fig. 6b). The clinical decision curve showed that when the threshold probability was between 2 and 78%, the model could be beneficial to estimating thrombosis risk (Fig. 6c). The clinical impact curve came from the clinical decision curve, which showed the estimated number of people at each risk threshold who would be declared high risk and visually showed the proportion of cases (true positive) (Fig. 6d).

**Figure 7:**
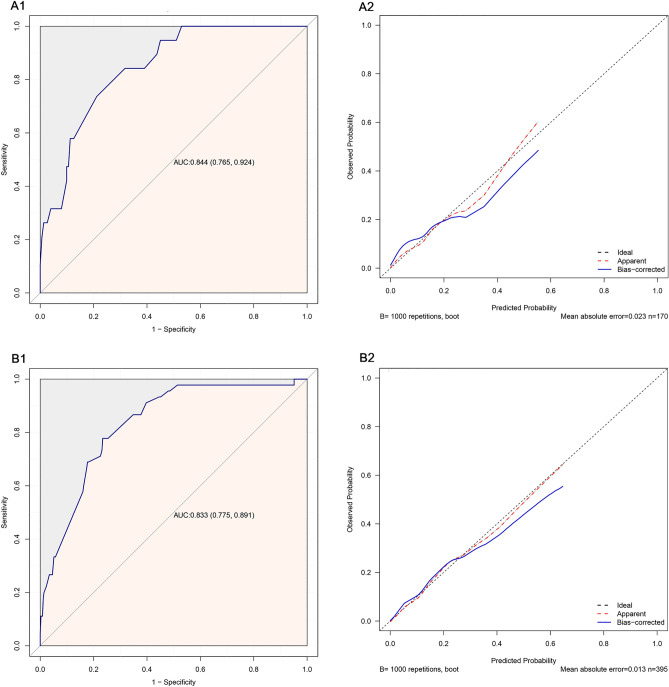
Internal validation of the final model. (**A1**) ROC curve for the model (30% of the study population). (**A2**) Calibration curve for the model (30% of the study population). (**B1**) ROC curve for the model (70% of the study population). (**B2**) Calibration curve for the model (70% of the study population).

Finally, we used Bootstrapping for internal validation (Fig. 7A1,A2/B1,B2). 30% and 70% of the complete data were re-sampled 1000 times for calculation. The AUC values were 0.844 (0.765, 0.924) and 0.833 (0.775, 0.891), respectively, and the calibration curves showed a good fit.

## Discussion

The influence factors of thrombosis are varied, such as damage to cardiovascular endothelial cells, changes in blood flow status (such as slowing of blood flow and turbulence), and increased blood coagulation^10,11^. Previous studies have found that in NIDCM patients, the intracardiac thrombosis is not only related to the patient’s heart cavity enlargement^12^ and reduced ventricular wall beat amplitude^13^ but also related to many potential risk factors such as endocardial injury caused by myocardial inflammation^14^, hypercoagulable state caused by systemic inflammation^15^, congestion caused by heart failure^16,17^, and turbulence caused by arrhythmia. All these factors lead to the activation of the coagulation system and eventually thrombosis. Cardiovascular intimal injury is the most important and common cause of thrombosis^10^.

Our study found that in the univariate analysis between groups, the indicators related to myocardial inflammation and myocardial damage showed statistical differences, such as hs-CRP, CK-MB, LDH, LD1, α-HBD, AST, and ALT. After variable selection, we finally screened out hs-CRP as a risk indicator. And the risk of thrombosis in patients with hs-CRP ≥ 1 mg/L was 3.1 times that of hs-CRP < 1 mg/L. In many previous studies, hs-CRP is an excellent diagnostic indicator for myocarditis^18^, and it has also been certified to be related to thrombosis^19–21^. Myocarditis is a recognized cause of NIDCM. In up to 30% of cases, biopsy-proven myocarditis can progress to NIDCM and is associated with a poor prognosis^22^. Evidence for the evolution of myocarditis to NIDCM comes from several sources, including animal models and patients^23^. Matsumori and Kawai^24^ established myocarditis models in an inbred line of DBA/2 mice infected with encephalomyocarditis (EMC) virus and observed progressive ventricular dysfunction and dilation. In addition, dilatation and hypertrophy of the heart persisted to the eighth month after inoculation with EMC virus in C3H/He and DBA/2 mice^25^. However, myocarditis is a challenging diagnosis due to the heterogeneity of clinical presentation. These patients may have been undiagnosed before developing NIDCM because endomyocardial biopsy is the gold standard for diagnosis and is rarely used^22^. This suggests that future studies should conduct imaging to determine whether myocardial inflammation and/or fibrosis are present in NIDCM patients with mural thrombi, which will be beneficial to understand the relationship between thrombosis and myocarditis. In addition, the exact etiology may be helpful to improve the accuracy of the nomogram.

The WBC is a marker of inflammation that is widely available in clinical practice. WBC adhesion to endothelium and platelets plays a vital role in the activation of coagulation. Excessive activation of WBC during inflammatory reactions may induce a systemic procoagulant state^26^. Therefore, when inflammation is present, the blood is usually in a hypercoagulable condition, thereby promoting the formation of a thrombus^27^. Thrombosis and inflammation are separate physiological processes yet an intense interdependence between these mechanisms has been recognized over the past decade, which may be related to immunothrombosis^28^. This study showed that elevated WBC was independently associated with an increased risk of thrombosis, suggesting that inflammation plays a significant role in thrombosis.

NT‐proBNP has widely used in clinical practice as a marker of heart failure. In our study, the risk of intracardiac thrombosis in patients with NT-proBNP ≥ 1800 pg/mL was about 6.0 times that of patients with NT-proBNP < 1800 pg/mL. We believe people with NT-proBNP ≥ 1800 pg/mL have a worse cardiac function. As myocardial contractility decreases and the expansion of the heart cavity, resulting in a change in the state of blood flow, which is more prone to thrombosis^29^. Actually, a variety of factors associated with heart failure predispose to thrombosis. The more severe the heart failure, the more likely the tissues to appear hypoxia and blood flow stasis, which lead to extensive endothelial damage, thereby contributing to thrombosis^29^. In addition, a study showed that NT-proBNP was a key predictor of heightened thrombin formation in AF, and the predictive value might be partly attributed to prothrombotic blood alterations^30^.

D-dimer serves as an important indicator of intravascular thrombosis^31^. In addition, elevated d-dimer following anticoagulation for a thrombotic event indicated an increased risk of recurrent thrombosis^32^. A long-term follow-up study involving 11,415 participants found that higher basal plasma D-dimer concentration was associated with a higher risk of ischemic stroke, especially cardiovascular stroke^33^. In our model, positive D-dimer was associated independently with intracardiac thrombosis. Hematocrit is defined as the volume ratio of red blood cells to whole blood, which indirectly reflects the number and volume of red blood cells. It is also a critical factor that affects blood viscosity. The univariate analyses suggested positive associations between HB and RDWCV with thrombosis, which supported that hemoconcentration was potentially associated with thrombosis. Pulse pressure refers to the difference between systolic pressure and diastolic pressure. As the heart function decreases, stroke volume reduces, and systolic pressure also decreases in patients with NIDCM. At the same time, the compensation mechanism of the heart increases the heart rate and the diastolic pressure. As a result, the pulse pressure would decrease. Our research suggested that arterial pressure and heart rate might be associated with intracardiac thrombosis, which also supported the role of hemodynamics in the development of thrombosis. In many studies, a history of stroke as an accessible indicator was often included in the thrombosis risk model, and our model was no exception. In addition, in multivariate analysis, we found atrial fibrillation was a key factor for thrombus in the left atrial, but not for thrombi elsewhere in the heart cavity. Moreover, in clinical work, the first concern is whether thrombosis will occur, which is directly related to the need for prophylactic medication, and the second concern is the location of thrombosis. So, we did not include that variable in the model. Nevertheless, the role of atrial fibrillation needs to be further studied, and more data is necessary.

In the past, people did not have a deep understanding of NIDCM complicated thrombosis and did not understand its incidence, etiology, and prognosis. With the deepening of understanding and the advancement of auxiliary examination technology, the detection rate of thrombosis is getting higher, making early detection and treatment possible^34,35^. In addition, treatment methods have become more selective, such as drugs, surgery, or interventional techniques^35^. However, NIDCM patients are often accompanied by severe cardiac insufficiency^36^. A large part of patients diagnosed for the first time have poor cardiac function, and cannot tolerate surgery or even some examinations. In addition, NIDCM has brought a huge economic burden to individuals, families, and society as a kind of chronic progressive disease. The costs of some high-tech methods are often beyond the reach of patients. Therefore, early identifying patients at high risk of thrombosis becomes particularly important, and early intervention can effectively prevent cardiovascular embolism events^37^.

We established this risk model based on accessible clinical indicators. The seven risk factors represented several dimensions that affected thrombosis. As these factors are easy to collect, the nomogram is not only suitable for inpatients but also outpatients. The nomogram is a useful supplementary tool for clinical work, and it has positive clinical significance in the decision-making process of diagnosis and treatment^38^. We know that in a typical DCM diagnosis and treatment process, echocardiography is usually part of the examination, which will facilitate the detection of intracardiac thrombus. Nevertheless, the diagnosis of thrombus is subject to certain subjectivity and complexity, and it requires a very skilled and experienced sonographer to make accurate judgments. Many grassroots hospitals do not have such medical conditions. Using a nomogram for risk assessment, it is still necessary for patients with a high risk of thrombosis to be referred to a higher-level medical center for re-examination. If necessary, transesophageal ultrasound or cardiac magnetic resonance can be performed to clarify the condition. In addition, for critically ill patients, the risk of going for an examination is extremely high, and bedside echocardiography is not routinely performed in many medical institutions. Even if conditions are available, the results are unreliable because the patients are usually unable to lie supine, and the images are often blurred due to the presence of lacunar effusion and tissue edema. At this point, the nomogram will be a useful auxiliary assessment tool. And the most crucial point is that even in a large and well-equipped medical center, imaging examinations can only answer the question of whether there is a thrombus, but cannot answer whether there is a high risk of thrombosis in the current state of the patient. This study comprehensively considered potential risk factors, adopted multiple methods to screen variables, and analyzed them in different subgroups. Finally, a concise model with good identification and calibration is constructed, which provides a useful reference for clinical risk assessment. However, there are also several limitations: Firstly, we obtained the datasets of our study from the clinical record of one hospital, which may not be sufficiently representative of all patients with NIDCM. In addition, potential risk factors were not considered thoroughly, such as ventricular sphericity and genetic variation. Secondly, though the robustness of our model was examined extensively with internal validation using bootstrap testing, the external validation of the current risk model was also important. Therefore, more studies are needed. We would try to persuade other medical centers to join this research project to conduct a more in-depth assessment and validation of the nomogram. Thirdly, it must be acknowledged that due to the cross-sectional design of this research, the role of the model is limited to estimating the immediate risk of thrombosis rather than a true prognosis that can inform long-term treatment decisions. In fact, intracardiac thrombus formation is a highly elusive complication of NIDCM. Owing to its asymptomatic nature, it is challenging to detect promptly. Often, it is only discovered during diagnostic procedures, such as echocardiography, when other complications (such as heart failure and atrial fibrillation) manifest. Thus, we must acknowledge that we cannot pinpoint the exact timing of thrombus formation. Although we have previously noted that cardiac magnetic resonance features can differentiate between acute and chronic thrombi^39^ (acute thrombi exhibit high signal intensity on T1 and T2-weighted images, while chronic thrombi show low signal intensity on T1 and T2 sequences, occasionally with signs of calcification), unfortunately, we lack complete data in this regard. We also cannot ascertain the extent to which the use of anticoagulants and antiplatelet drugs before admission has impacted thrombus formation and related variables, although this subset of patients constitutes a very small proportion. Cross-sectional data also imply that we are unaware of whether the variables (side effects of thrombus formation) in the risk model are predictive factors for future risks. For instance, ‘D-dimer, white blood cell count, and high-sensitivity C-reactive protein’ may represent coagulation cascade activation and subsequent mild inflammatory response during thrombus formation. These variables are likely to be consequences rather than causes of the thrombus formation process. However, whether they are causes or consequences, their correlation is evident. Furthermore, the models developed in this research lack validation of the prospective patient outcomes determined after diagnosis. Consequently, prospective cohort studies are still required to disclose causal links and further predict future thrombosis risk, and guide prevention. Nonetheless, we understand that thrombus formation is generally closely related to the state of the body at that time. In longitudinal cohort studies, these predictors may change over time due to changing body conditions, leading to chance in results. Therefore, the cross-sectional design appears to be better at screening for the factors most strongly associated with intracardiac thrombotic events. These factors explain the formation of thrombus from multiple dimensions, which is valuable to assessing the thrombosis risk of DCM patients as a whole. In addition, this study also provides a basis and useful reference for subsequent research.

## Conclusions

The current study constructed an individualized intracardiac thrombosis risk estimation model for NIDCM patients with good discrimination and calibration. The model also performed well in different thrombosis distributions. To the best of our knowledge, this is the first nomogram for the risk of intracardiac thrombosis among NIDCM patients. The probability of intracardiac thrombosis can be easily estimated through the nomogram, which will help improve the early identification and screening of intracardiac thrombosis among NIDCM patients. In addition, Atrial fibrillation may be a critical risk factor for left atrial thrombosis in NIDCM patients, but more data is needed to support this conclusion.

### Supplementary Information

Supplementary Information.

## Data Availability

The datasets used and/or analyzed during the current study are available from the corresponding author on reasonable request.

## Abbreviations

AF: Atrial fibrillation
A/G: ALB/GLOB
α-HBD: Alpha-hydroxybutyrate dehydrogenase
ALT: Alanine aminotransferase
ALB: Albumin
APTT: Activated partial thromboplastin time
AST: Aspartate aminotransferase
AUC: Area under the curve
BIC: Bayesian Information Criterion
CI: Confidence interval
CK: Creatine kinase
CK-MB: Creatine kinase-MB
CO: Cardiac output
CysC: Cystatin c
DCA: Clinical decision curve
EMC: Encephalomyocarditis
FIB: Fibrinogen
GLOB: Globulin
HB: Hemoglobin
Hcy: Homocysteine
HDL: High density lipoprotein
hs-CRP: High-sensitivity c-reactive protein
ICM: Ischemic cardiomyopathy
IDI: Integrated discrimination improvement
LAD: Left atrium anteroposterior dimension
LASSO: Least absolute shrinkage and selection operator
LDH: Lactic dehydrogenase
LD1: Lactic dehydrogenase-1
LDL: Low-density lipoprotein
LVDd: Left ventricular end diastolic dimension
LVDs: Left ventricular end systolic dimension
LVFS: Left ventricular fractional shortening
LVEF: Left ventricular ejection fraction
LVT: Left ventricular thrombosis
NEU%: Percentage of neutrophils
NIDCM: Non-ischemic dilated cardiomyopathy
NLR: Neutrophil to lymphocyte ratio
NRI: Net reclassification improvement
NT-proBNP: N terminal pro B type natriuretic peptide
OR: Odds ratio
RBC: Red blood cell count
RDWCV: Red blood cell volume distribution width
ROC: Receiver operating characteristic curves
SD: Standard deviation
SV: Stroke volume
TC: Total cholesterol
TG: Triglycerides
TT: Thrombin time
VIFs: Variance inflation factors
WBC: White blood cell count
