# Author Correction: Development and validation of a risk model for intracardiac thrombosis in patients with dilated cardiomyopathy: a retrospective study

**DOI:** 10.1038/s41598-024-59433-5

**Published:** 2024-04-16

**Authors:** Yuan Huang, Long-Chang Li, Yu-Xin Li, Chun Gui, Li-Hua Yang

**Affiliations:** 1 Department of Cardiology, Jiangbin Hospital of Guangxi Zhuang Autonomous Region, Nanning, Guangxi China; 2 Department of Cardiology, The First People’s Hospital of Nanning, The Fifth Affiliated Hospital of Guangxi Medical University, Nanning, Guangxi China; 3 Department of Cardiology, The First Affiliated Hospital of Guangxi Medical University, Nanning, 530021 Guangxi China; 4 Guangxi Key Laboratory Base of Precision Medicine in Cardio-Cerebrovascular Diseases Control and Prevention, Nanning, 530021 Guangxi China; 5 Guangxi Clinical Research Center for Cardio-Cerebrovascular Diseases, Nanning, 530021 Guangxi China

Correction to: *Scientific Reports* 10.1038/s41598-024-51745-w, 16 January 2024

The original version of this Article contained an error in the order of the author names, which was incorrectly given as Yuan Huang, Long-Chang Li, Yu-Xin Li, Li-Hua Yang & Chun Gui.

The original Article has been corrected.

