# Supplementary material for: Development and validation of a risk model for intracardiac thrombosis in patients with dilated cardiomyopathy: a retrospective study
Source: Sci Rep. 2024 Jan 16;14:1431. doi: 10.1038/s41598-024-51745-w (PMC10791606; doi:10.1038/s41598-024-51745-w)

Supplementary material

Supplementary Table 1. The best cutoff point value of the five continuous variables.

variable	AUC	Youden Index(maximum)	Cut-off point(corresponding)	Cut-off point(final)
D-dimer	0.719	0.385	484ng/ml	450ng/ml
NT-proBNP	0.625	0.240	2085.5pg/ml	1800pg/ml
WBC	0.629	0.244	7.4×10^9/L	10×10^9/L
Pulse pressure	0.617	0.252	45mmHg	40mmHg
Hematocrit	0.624	0.216	0.43	<0.4,0.4-0.5,≥0.5

Notes: Receiver-operating characteristic (ROC) curve analysis was performed to determine the optimal cutoff point value of the five indexes.

Abbreviations: WBC,white blood cell count; AUC,area under the curve.

Supplementary Table 2: The distribution of thrombus in the heart cavity(67cases in total)

Notes: Among 67 patients with dilated cardiomyopathy with thrombosis, most of them were mural thrombus (66 cases, accounting for 98.5%), and there were very few free thrombus (1 case, accounting for 1.5%). In addition, single thrombosis was the main one (46 cases, 68.7%), and multiple thrombosis was relatively rare (21 cases, 31.3%). The thrombus was located in the left ventricle (52 cases, accounting for 77.6%), left atrium (1 case, accounting for 1.5%), left atrial appendage (14 cases, accounting for 20.9%) and right ventricle (6 cases, accounting for 9.0%); In the ventricle, thrombus at the apex accounted for the highest proportion (37 cases, 71.2%). In terms of mobility, most of the thrombus did not change position with the contraction of the heart, and a small part of the thrombus tail could swing accordingly. Only 1 case did not see the attachment point and was in a free state.

Number	Attachment point	Quantity	Size(Long axis×short axis)	Mobility
1	Left ventricular free wall muscle trabecular crypt	>3	Too small to measure	stable
2	Left atrial appendage	1	18mm×11mm	stable
3	Left atrial appendage	1	9mm×9mm	stable
4	Left ventricular apex	1	18mm×9mm	swing
5	Left ventricular free wall muscle trabecular crypt	>3	Too small to measure	stable
6	Left atrial appendage	1	10mm×9mm	stable
7	Left ventricular free wall muscle trabecular crypt	>3	Too small to measure	stable
8	Anterior compartment of left ventricle	1	14mm×8mm	stable
9	Left ventricular apex	3	17mm×15mm、14mm×13mm、12mm×11mm	stable
10	Left ventricular apex	1	21mm×10mm	stable
	Left ventricular free wall muscle trabecular crypt	>3	Too small to measure	stable
11	Left ventricular apex	1	8mm×6mm	swing
12	Right ventricle	1	16mm×11mm	stable
	Left atrial appendage	1	10mm×13mm	stable
13	Left atrium (no attachment)	1	24mm×19mm	free
14	Left ventricular free wall muscle trabecular crypt	>3	Too small to measure	stable
15	Posterior wall of left ventricle	1	14mm×9mm	swing
16	Left ventricular apex	1	19mm×15mm	swing

**17**	Left ventricular apex	2	50mm×27mm、24mm×10mm	swing
**18**	Left ventricular apex	1	33mm×18mm	stable
**19**	Left ventricular apex	1	29mm×18	stable
**20**	Left ventricular apex	2	30mm×15mm、26mm×10mm	stable
	Right ventricular apex	1	26mm×18mm	stable
**21**	Left ventricular apex	1	45mm×36mm	swing
**22**	Left ventricular apex	1	28mm×12mm	stable
**23**	Left atrial appendage	1	12mm×7mm	stable
**24**	Anterior wall of left ventricle	1	47mm×10mm	stable
**25**	Left ventricular apex	1	38mm×12mm	stable
**26**	Left ventricular free wall muscle trabecular crypt	>3	Too small to measure	stable
**27**	Left atrial appendage	1	15mm×10mm	swing
**28**	Left atrial appendage	1	12mm×7mm	stable
**29**	Right side wall of right ventricle	1	21mm×17mm	stable
**30**	Left ventricular apex	1	53mm×44mm	stable
	Right ventricular apex	1	31mm×17mm	stable
**31**	Left ventricular apex	1	71mm×10mm	stable
	Right ventricular apex	1	46mm×23mm	stable
**32**	Left atrial appendage	1	18mm×13mm	stable
**33**	Left ventricular apex	1	23mm×13mm	swing
**34**	Left ventricular apex	1	26mm×17mm	stable
**35**	Left ventricular apex	1	32mm×27mm	swing
	Left atrial appendage	1	20mm×13mm	stable
**36**	Left ventricular apex	1	23mm×15mm	stable
**37**	Left ventricular septum	1	15mmx11mm	stable
**38**	Left ventricular apex	1	14mm×8mm	swing
**39**	Left ventricular	>3	35mm×18mm、8mm×16mm（the larger）	stable
**40**	Left ventricular	1	54mm×35mm	stable
**41**	Left ventricular apex	1	33mm×18mm	stable
**42**	Tip of left atrial appendage	1	11mm×8mm	stable
**43**	Left ventricular apex	1	18mm×14mm	stable
**44**	Tip of left atrial appendage	1	23mm×13mm	stable
**45**	Tip of left atrial appendage	1	39mm×27mm	stable
**46**	Side wall of left ventricle	1	40mm×17mm	swing
**47**	Left ventricular apex	2	12mm×11mm、14mm×12mm	swing
**48**	Left ventricular apex	1	22mm×16mm	swing
**49**	Tip of left atrial appendage	1	16mm×11mm	stable
**50**	Left ventricular apex	2	23mm×12mm、26mm×10mm	stable
**51**	Left ventricular apex	1	35mm×24mm	swing

	Left atrial appendage	>3	14mm×11mm（the larger）	stable
**52**	Right ventricular apex	1	21mm×16mm	stable
**53**	Left ventricular apex	1	31mm×9mm	stable
**54**	Left ventricular	>3	39mm×19mm（the larger）	swing/stable
**55**	Anterior wall of left ventricle	1	36mm×12mm	stable
**56**	Left ventricular septum	3	54mm×19mm、15mm×14mm、16mm×11mm	stable
**57**	Left ventricular apex	1	38mm×25mm	swing
**58**	Left ventricular apex	1	40mm×12mm	stable
**59**	Left ventricular apex	1	41mm×22mm	stable
**60**	Left ventricular apex	1	52mm×15mm	stable
**61**	Left ventricular apex	1	50mm×21mm	stable
**62**	Left ventricular apex	1	16mm×12mm	stable
**63**	Left ventricular apex	1	23mm×21mm	stable
**64**	Left ventricular apex	1	43mm×22mm	stable
**65**	Left ventricular apex	1	30mm×24mm	stable
**66**	Left ventricular apex	>3	27mm×20mm、19mm×18mm、20mm×14mm（the larger）	stable
**67**	Left ventricular apex	2	30mm×16mm、15mm×12mm	stable
	Anterior wall of left ventricle	1	29mm×15mm	swing

Supplementary Table 3: Baseline characteristics of cohort population and excluded population(insufficient data).

Variables	Cohort population (n = 564)	Excluded population (n=186)	Z/χ^2	P-value
Thrombosis , n (%)			0.235	0.628
No	497 (88.1)	167 (89.8)		
Yes	67 (11.9)	19 (10.2)		
**General information**				
Gender , n (%)			5.265	**0.022**
Female	124 (22.0)	57 (30.6)		
Male	440 (78.0)	129 (69.4)		
Age of onset, n (%)			0.494	0.781
<45years	170 (30.1)	55 (29.6)		
45-65years	314 (55.7)	108 (58.1)		
≥65years	80 (14.2)	23 (12.4)		
Medical history, n (%)			0.923	0.63
<1 year	273 (48.7)	83 (44.6)		
1-5 years	183 (32.6)	66 (35.5)		
≥5 years	105 (18.7)	37 (19.9)		
Smoking history, n (%)			5.317	**0.021**
No	295 (52.3)	116 (62.4)		
Yes	269 (47.7)	70 (37.6)		
Drinking history, n (%)			6.171	**0.013**
No	300 (53.2)	119 (64)		
Yes	264 (46.8)	67 (36)		

Hypertension, n (%)			1.69	0.194
No	463 (82.1)	161 (86.6)		
Yes	101 (17.9)	25 (13.4)		
Pulmonary hypertension, n (%)			0.201	0.654
No	191 (33.9)	67 (36)		
Yes	373 (66.1)	119 (64)		
Atrial fibrillation, n (%)			0.414	0.52
No	454 (80.5)	145 (78)		
Yes	110 (19.5)	41 (22)		
History of stroke, n (%)			0.584	0.445
No	509 (90.2)	172 (92.5)		
Yes	55 (9.8)	14 (7.5)		
Diabetes, n (%)			0.158	0.691
No	473 (83.9)	153 (82.3)		
Yes	91 (16.1)	33 (17.7)		
Grade of heart failure(NYHA), n(%)			1.55	0.671
I	20 (3.5)	7 (3.8)		
II	98 (17.4)	36 (19.4)		
III	214 (37.9)	76 (40.9)		
IV	232 (41.1)	67 (36)		
**Physical examination**				
Body mass index(㎏/㎡)	23.0 (20.5, 25.2)	23.3 (21.5, 25.7)	1.523	0.217
Heart rate(times/min)	89.0 (77.0, 102.0)	84.0 (74.0, 96.0)	5.297	**0.021**
Systolic pressure(mmHg)	112.0 (102.0, 128.0)	114.0 (102.2, 125.5)	0.261	0.61
Diastolic pressure(mmHg)	75.0 (66.0, 86.0)	76.0 (68.0, 83.8)	0.006	0.939
Pulse pressure				
-Measurement data(mmHg)	37.0 (29.8, 49.0)	37.0 (30.0, 45.0)	0.372	0.542
-Count data(%)			0.13	0.719
<40mmHg	320 (56.7)	109 (58.6)		
≥40mmHg	244 (43.3)	77 (41.4)		
**Blood biochemical**				
NT-proBNP				
-Measurement data(pg/ml)	3998(1987, 8325)	3210.0 (1411.0, 6584.0)	4.781	**0.029**
-Count data(%)			0.272	0.602
<1800pg/ml	127 (22.5)	46 (24.7)		
≥1800pg/ml	437 (77.5)	140 (75.3)		
hs-CRP(%)			Fisher	0.785
<1mg/L	118 (20.9)	5 (23.8)		
≥1mg/L	446 (79.1)	16 (76.2)		
WBC				
-Measurement data($\times 10^9$/L)	7.5 (6.2, 9.1)	7.7 (6.3, 9.4)	1.774	0.183
-Count data(%)			0.156	0.693
<10×10^9/L	467 (82.8)	157 (84.4)		
≥10×10^9/L	97 (17.2)	29 (15.6)		
RBC($\times 10^{12}$/L)	4.7 (4.3, 5.1)	4.7 (4.2, 5.2)	0.095	0.758
HB(g/L)	136.0 (125.0, 148.0)	136.6 (123.0, 148.3)	0.035	0.853
Platelet($\times 10^9$/L)	197.5 (162.8, 250.0)	197.8 (161.2, 241.5)	0.097	0.756

NEU%	0.6 (0.6, 0.7)	0.6 (0.6, 0.7)	3.3372	0.068
NLR	2.6 (1.8, 4.0)	2.3 (1.7, 3.7)		
RDWCV	0.2 (0.1, 0.2)	0.1 (0.1, 0.2)		
Hematocrit				
-Measurement data	0.4 (0.4, 0.5)	0.4 (0.4, 0.5)	1.556	0.212
-Count data(%)			3.145	0.208
<0.4	192 (34.0)	65 (35.3)		
0.4-0.5	342 (60.6)	103 (56)		
≥0.5	30 (5.3)	16 (8.7)		
APTT(s)	32.0 (30.2, 33.8)	31.4 (29.9, 34.6)	0.278	0.598
FIB(g/L)	3.8 (3.0, 4.1)	3.8 (3.1, 4.6)	6.694	**0.01**
TT(s)	12.0 (11.3, 12.5)	11.9 (11.0, 12.8)	0.046	0.83
D-dimer(%)			Fisher	1
Negative(<450ng/ml)	315 (55.9)	0 (NaN)		
Positive(≥450ng/ml)	249 (44.1)	0 (NaN)		
CK(U/L)	91.0 (61.0, 147.0)	80.5 (54.0, 125.2)	5.063	**0.024**
CK-MB(U/L)	15.0 (11.0, 21.0)	15.0 (12.0, 19.0)	0.136	0.712
LDH(U/L)	261.0 (207.0, 327.5)	244.5 (209.0, 303.0)	1.658	0.198
LD1(U/L)	71.0 (55.0, 95.2)	65.0 (41.5, 86.8)	12.821	**< 0.001**
α-HBD(U/L)	191.0 (148.0, 230.0)	181.0 (152.0, 221.0)	1.505	0.22
TC(mmol/L)	4.2 (3.5, 4.9)	4.3 (3.6, 5.1)	1.393	0.238
TG(mmol/L)	1.0 (0.8, 1.4)	1.1 (0.8, 1.6)	4.428	**0.035**
HDL(mmol/L)	1.0 (0.8, 1.2)	1.1 (0.8, 1.3)	9.383	**0.002**
LDL(mmol/L)	2.6 (2.0, 3.2)	2.6 (2.1, 3.2)	0.506	0.477
Hcy(μmol/L)	15.2 (11.9, 18.3)	15.9 (12.4, 19.4)	2.421	0.12
K^+ (mmol/L)	4.0 (3.7, 4.3)	4.1 (3.8, 4.4)	2.673	0.102
Na^+(mmol/L)	139.0 (136.0, 141.0)	139.5 (137.2, 141.5)	2.251	0.133
Cl^-(mmol/L)	102.0 (99.0, 106.0)	102.2 (98.6, 105.4)	0.025	0.874
ALB(g/L)	38.2 (35.2, 41.2)	39.1 (36.0, 42.0)	4.744	**0.029**
GLOB(g/L)	26.2 (23.0, 30.0)	26.0 (23.1, 29.4)	0.087	0.768
A/G	1.5 (1.3, 1.7)	1.5 (1.3, 1.8)	3.837	0.05
AST(U/L)	32.0 (23.0, 47.0)	28.0 (21.5, 39.0)	8.642	**0.003**
ALT(U/L)	31.0 (19.0, 55.0)	29.0 (19.5, 47.5)	0.505	0.477
UREA(mmol/L)	7.1 (5.4, 9.1)	6.8 (5.6, 9.0)	0.265	0.607
Creatinine(μmol/L)	93.5 (79.0, 114.0)	90.0 (76.0, 115.0)	2.387	0.122
CysC(mg/L)	1.1 (0.8, 1.3)	1.1 (0.9, 1.3)	0.208	0.649
UA(μmol/L)	495.0 (391.0, 630.0)	476.0 (361.8, 584.0)	4.147	**0.042**
**Echocardiographic**				
LAD(mm)	46.0 (41.0, 51.0)	46.0 (41.2, 50.0)	0.02	0.888
LVDd(mm)	69.0 (64.0, 75.0)	72.0 (64.0, 78.0)	4.888	**0.027**
LVDs(mm)	58.0 (53.0, 63.0)	59.0 (52.0, 65.8)	0.558	0.455
LVFS(%)	16.0 (13.0, 19.0)	17.0 (14.0, 20.0)	5.638	**0.018**
LVEF(%)	33.0 (26.0, 39.0)	34.0 (29.0, 40.8)	4.559	**0.033**
SV(ml/B)	80.0 (61.0, 98.0)	88.0 (67.2, 111.6)	10.519	**0.001**
CO(L/min)	7.1 (5.2, 8.9)	7.5 (5.5, 9.9)	5.573	**0.018**

Note: P value < 0.05 is marked in red.

Supplementary Table 4: Logistic regression results of the model S/L-LAT (LAT data).

Variable	Unadjusted OR (95% CI)	Unadjusted P-value	Adjusted OR (95% CI)	Adjusted P-value
(Intercept)	0.02 (0.01~0.03)	<0.001	0 (0~Inf)	0.991
AF	6.19 (2.15~17.79)	0.001	5.59 (1.78~17.5)	0.003
NT-proBNP	34457073.31 (0~Inf)	0.991	53671586.71 (0~Inf)	0.994
Pulse pressure	0.29 (0.08~1.02)	0.054	0.3 (0.08~1.14)	0.078
hs-CRP	12344215.28 (0~Inf)	0.987	26154301.83 (0~Inf)	0.994
WBC	8.44 (2.92~24.41)	<0.001	7.54 (2.4~23.65)	0.001
A/G	0.24 (0.05~1.14)	0.072	0.26 (0.05~1.42)	0.119

Model S/L-LAT (LAT data): Probability(Left atrial thrombus)=1/1+exp-[-37.037+(AF×1.721)+(NT-proBNP×17.798)-(Pulse pressure×1.211)+(hs-CRP×17.08)+(WBC×2.02)-(A/G×1.363)].

Supplementary Table 5: Comparison of model S/L-NAF with baseline model in terms of AUC, NRI and IDI.

Index	Model S/L-NAF[a] VS Baseline model[b]
AUC [95% CI],	0.843[0.789-0.897] VS 0.846[0.791-0.901],
p-value	0.454
NRI(Categorical) [95% CI],	0.020[-0.035-0.074],
p-value	0.480
NRI^+	0.037
NRI^-	-0.017
NRI(Continuous) [95% CI],	0.149[-0.097-0.394],
p-value	0.236
IDI [95% CI],	0.005[-0.005-0.016],
p-value	0.331

Variables included in each model are as follows: [a]**Model S/L-NAF:** Hematocrit, hs-CRP, NT-proBNP, Pulse pressure, History of stroke, D-dimer. [b]**Baseline model:**Hematocrit, WBC, hs-CRP, NT-proBNP, Pulse pressure, History of stroke, D-dimer.

Supplementary Figure 1. Comparison between the complete model(model S/L-LAT) and the model without A/G.

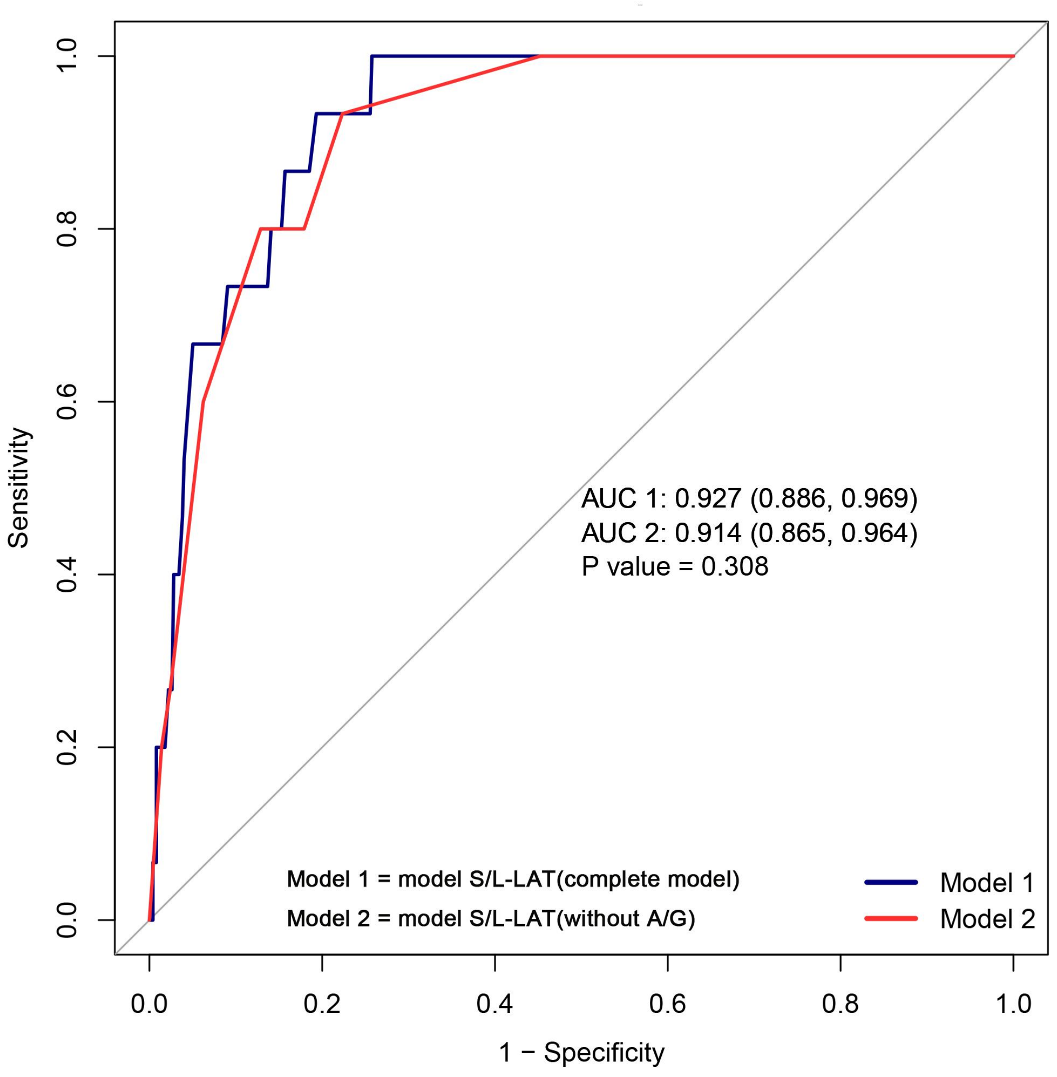

Supplementary Figure 2. Comparison between the complete model(model S/L-LAT) and the model without AF.

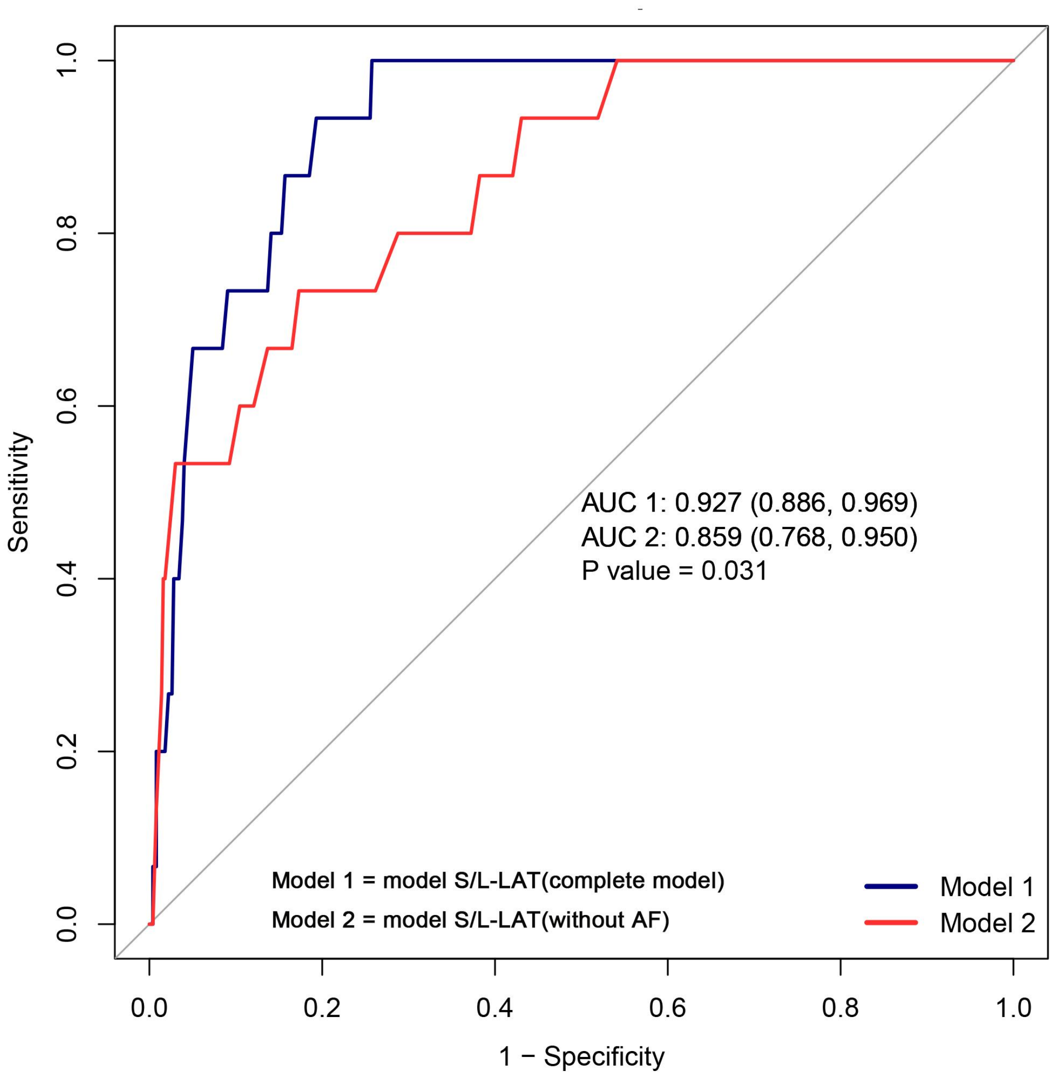

Supplementary Figure 3. ROC curve of baseline model in non-AF population.

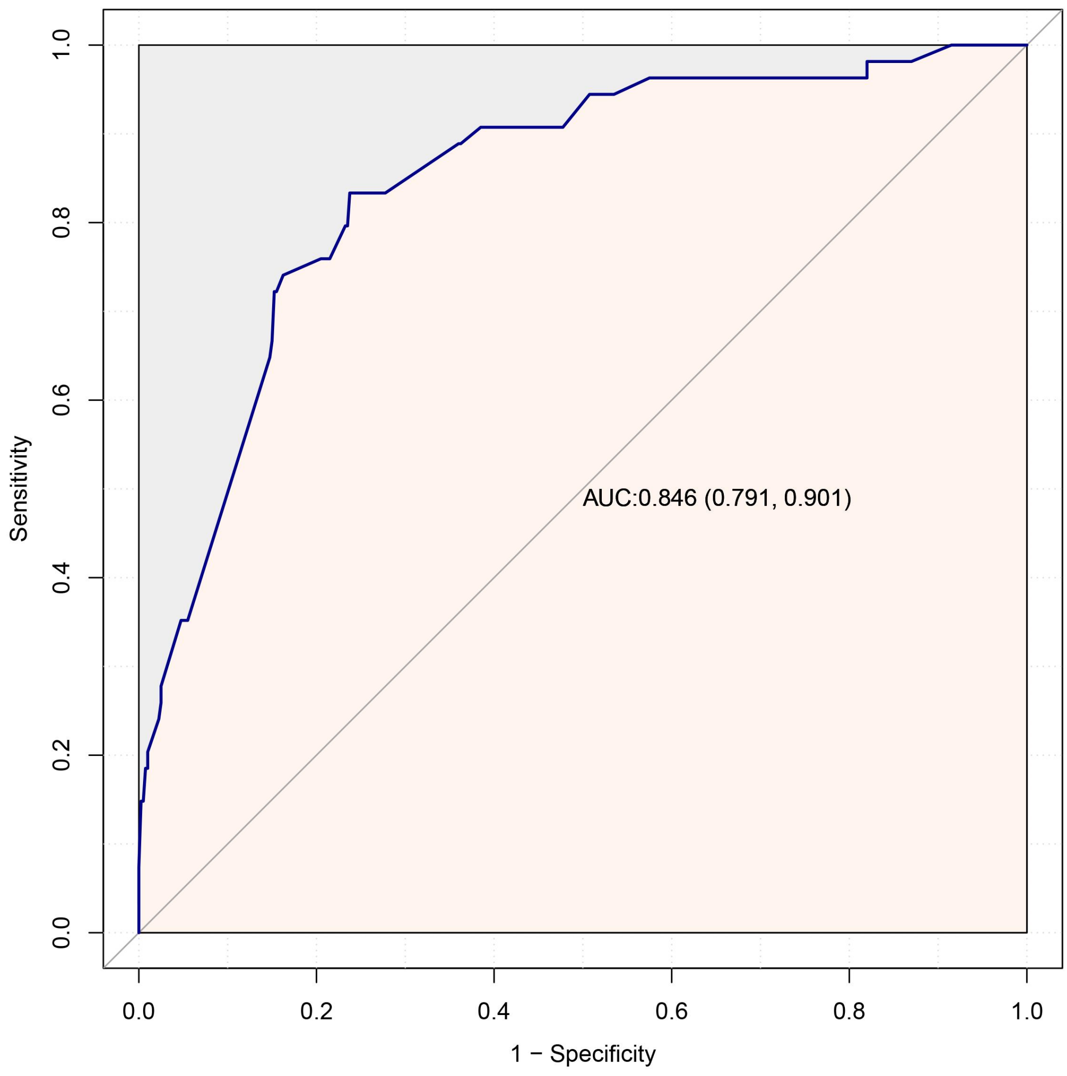

Supplementary Figure 4. Calibration curve of the baseline model in the non-AF population.

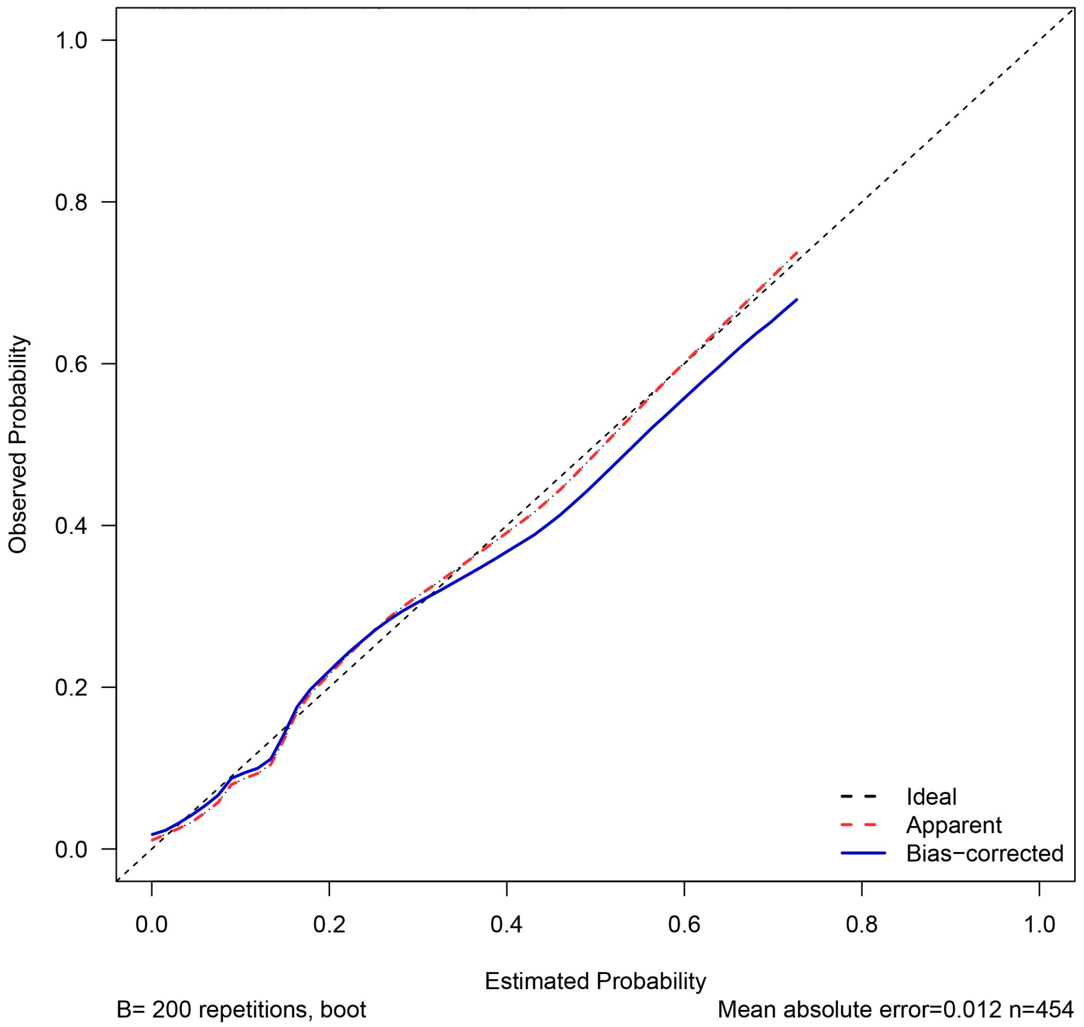

Supplement: Supplementary file 1 — Supplementary Information. [file 41598_2024_51745_MOESM1_ESM.pdf]
